# Phase I/II trial of lenalidomide, methotrexate, leucovorin, cytarabine, and rituximab (LeMLAR) in relapsed or refractory diffuse large B cell lymphoma

**DOI:** 10.1038/s41408-021-00485-5

**Published:** 2021-05-17

**Authors:** Ulrich Dührsen, Mareike Tometten, Frank Kroschinsky, Arnold Ganser, Stefan Ibach, Stefanie Bertram, Andreas Hüttmann

**Affiliations:** 1 Klinik für Hämatologie, Universitätsklinikum Essen, Essen, Germany; 2 Klinik für Hämatologie, Onkologie, Hämostaseologie und Stammzelltransplantation, Medizinische Fakultät, RWTH Aachen Universität, Aachen, Germany; 3 Medizinische Klinik und Poliklinik I, Universitätsklinikum Carl Gustav Carus, Dresden, Germany; 4 Klinik für Hämatologie, Hämostaseologie, Onkologie und Stammzelltransplantation, Medizinische Hochschule Hannover, Hannover, Germany; 5 X-act Cologne Clinical Research GmbH, Köln, Germany; 6 Institut für Pathologie, Universitätsklinikum Essen, Essen, Germany

**Keywords:** Phase I trials, Phase II trials, B-cell lymphoma, Chemotherapy, Cancer immunotherapy

Dear Editor,

Lenalidomide has moderate activity in relapsed or refractory diffuse large B cell lymphoma (r/rDLBCL)^1^. Based on the COMLA regimen used in the 1970s and 1980s^2^, we combined lenalidomide with methotrexate (plus leucovorin), cytarabine, and rituximab (LeMLAR) in a phase I/II trial. Lenalidomide induces apoptosis and cell cycle arrest in the G0/G1 phase^3^. After discontinuation, surviving cells start to proliferate and become susceptible to the S-phase-specific agents methotrexate and cytarabine administered in the subsequent cycle. Because of low toxicity for immune effector cells^4–6^, these drugs are not expected to incapacitate lenalidomide’s indirect mechanisms of action^3^.

The study was approved by the ethics committees of the participating sites and registered under EudraCT no. 2012-001891-13 and ClinicalTrials.gov identifier NCT01788189. The trial protocol is provided in the Supplementary Information. In brief, patients 18 years of age or older with relapsed or refractory, biopsy-proven CD20-positive aggressive B cell lymphoma (excluding mantle cell lymphoma and central nervous system involvement) and a performance status of 0–3 were eligible. Exclusion criteria included inadequate organ function unrelated to lymphoma (neutrophils <1.0 /nl, platelets <75 /nl, creatinine clearance <60 ml/min, bilirubine ≥2.5 mg/dl, serum aspartate [AST] or alanin aminotransferase [ALT] >4× upper limit of normal [ULN]), active hepatitis B or C, human immunodeficiency virus infection, any other uncontrolled infection, as well as pregnancy and nursing period. All patients gave written informed consent. The LeMLAR regimen (Fig. 1) comprised up to six 28-day cycles consisting of lenalidomide (day 1–21), methotrexate (5–10 min i.v. bolus; day 1, 8, 15), leucovorin (four oral 45 mg doses six hours apart, starting 24 h after methotrexate), cytarabine (5-10 min i.v. bolus; day 1, 8, 15), and rituximab (375 mg/m², day 1). All patients received prophylactic enoxaparin (40 mg s.c.).

**Fig. 1:**
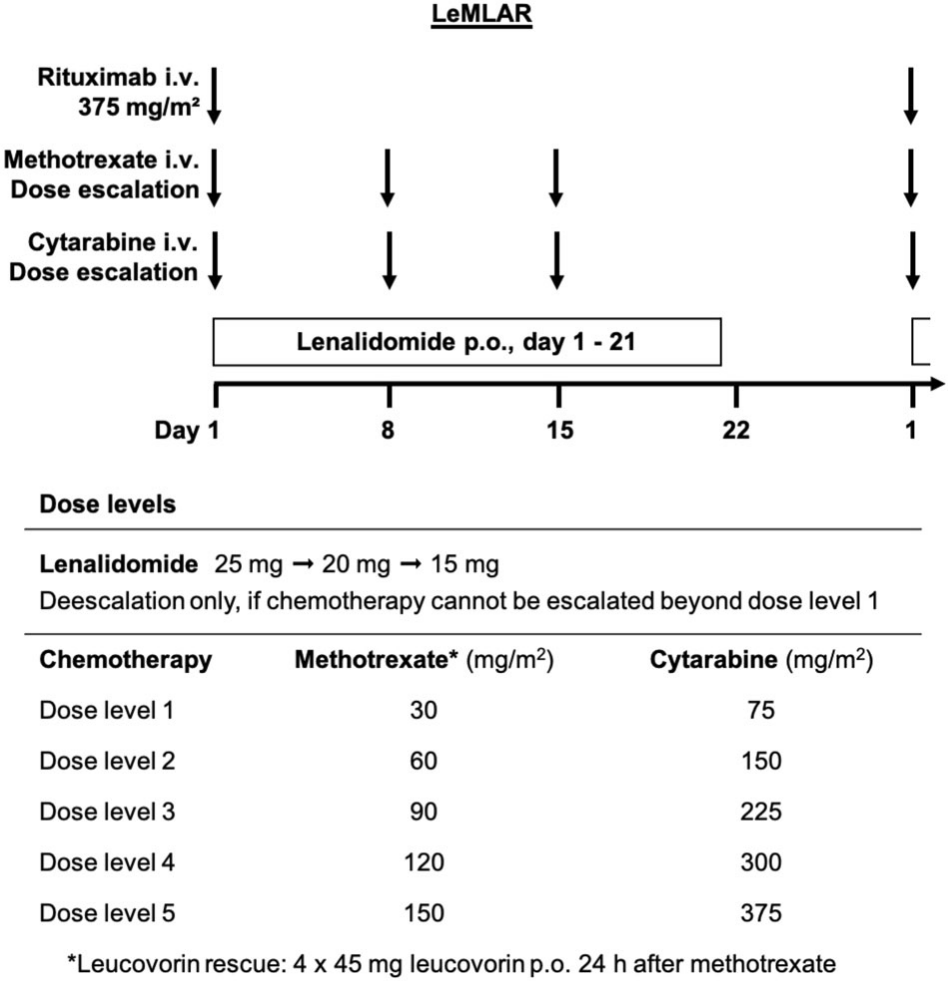
The LeMLAR protocol. Upper part: Treatment schedule (i.v., intravenous; p.o., per os). Lower part: Dose levels for maximum-tolerated-dose determination and intrapatient dose escalation.

In phase I, methotrexate and cytarabine were simultaneously escalated in a 3 + 3 design in 30 mg/m² or 75 mg/m² steps, respectively. The lenalidomide dose was reduced if chemotherapy could not be escalated beyond level 1. The maximum tolerated dose (MTD) was determined in cycles 1 and 2. Patients without dose-limiting toxicity (DLT) terminating treatment in cycle 1 or 2 prematurely due to disease progression were replaced by new patients. DLT was defined as neutrophils <0.5 /nl, platelets <25 /nl, creatinine clearance <60 ml/min, bilirubine ≥3 mg/dl, serum AST or ALT ≥6× ULN, or mucositis grade ≥3 on day 8 (plus a maximum of 3 extra days), day 15 (plus ≤6 days), or day 29 (plus ≤7 days). Failure to reach these thresholds at the indicated time-points prevented timely administration of methotrexate and cytarabine. Adverse events requiring dose reduction in cycle 1 or 2, receipt of <21 lenalidomide doses per cycle, cycle length >35 days, and any adverse event preventing continuation according to the protocol were also rated as DLT. Treatment tolerance was assumed if none of three or only one of six patients experienced DLT. In the absence of DLT, stepwise intrapatient dose escalation was possible after cycle 2, and again after cycle 4, in both phase I and phase II (Fig. 1). Sample size calculation in phase II was based on a reported objective response rate (ORR) to lenalidomide alone of 20%^7^. To demonstrate an ORR to LeMLAR of ≥40% at a two-sided α of 0.05 and a power of 0.4, 20 patients (including 10% drop-outs) were required to be treated at the MTD. Responses were assessed after the second and four weeks after the last cycle, using the international response criteria for malignant lymphomas^8^.

Between January 2013 and May 2018, 37 patients were registered from four academic sites. One patient withdrew consent and two were excluded by their physicians before treatment inception, leaving a total of 34 participants, with a median of 2 prior lines of therapy (range, 1–4). Twenty-two patients were recruited in phase I (Table 1). With a starting dose of 25 mg lenalidomide, chemotherapy level 1 was well tolerated. On level 2, two of three patients had a DLT (ALT increase, septicemia). After reduction of lenalidomide to 20 mg, one DLT was observed among six patients on level 2 (neutro-/thrombocytopenia), and two among six patients on level 3 (rash, mucositis). Thus, the MTD was level 2 (lenalidomide 20 mg, methotrexate 60 mg/m^2^, cytarabine 150 mg/m^2^).

**Table 1:** Patient characteristics.

	Phase I		Phase II	
	Number	Percent	Number	Percent
Number of patients	22	100%	20	100%
Diagnosis				
Diffuse large B cell lymphoma	21	95%	20	100%
Follicular lymphoma grade 3b	1	5%	–	–
Months from diagnosis, median (range)	13 (5–160)	n.a.	20 (3–89)	n.a.
Patient features				
Male	14	64%	11	55%
Age (years), median (range)	68 (47–81)	n.a.	62 (39–82)	n.a.
Age >60 years	15	68%	11	55%
ECOG performance status ≥2	9	41%	0	0%
Ann Arbor stage III or IV	19	86%	13	65%
≥2 extranodal lesions	17	77%	9	45%
Lactate dehydrogenase >ULN	18	82%	11	55%
B symptoms	3	14%	0	0%
International prognostic index				
Low	1	5%	6	30%
Intermediate-low	4	18%	5	25%
Intermediate-high	5	23%	6	30%
High	12	54%	3	15%
Previous therapies^a^				
1 prior line	7	32%	3	15%
2 prior lines	5	23%	11	55%
3 prior lines	7	32%	5	25%
4 prior lines	3	13%	1	5%
Autologous stem cell transplantation	4	18%	6	30%
Allogeneic stem cell transplantation	1	5%	1	5%
Refractory to last treatment	17	77%	13	65%
Relapse after last treatment	5	23%	7	35%

*ECOG* Eastern Cooperative Oncology Group, *n.a.* not applicable, *ULN* upper limitof normal.

Note that eight patients from the phase I were also included in phase II.

^a^See Supplementary Table 1 for regimens.

Phase II comprised eight MTD patients from the phase I and 12 new patients (Table 1). After two cycles, the ORR was 55%. Five patients achieved a complete remission (CR), six a partial remission (PR; one converting into CR on continued therapy), three had stable disease (SD), four progressive diseases (PD), and two were not evaluable (a receipt of only one cycle). The median duration of response (DOR) was 19.4 months, with four long-term survivors maintaining their responses for >21–32 months (Supplementary Fig. 1). Responses were seen in all of eight patients without and in three of 12 patients with progression on last treatment (median DOR to last treatment, 9.5 months, range 1–32; Supplementary Table 2). With a median follow-up of 31 months, median progression-free survival (PFS) and overall survival (OS) were 6.9 and 12.6 months, respectively (Supplementary Fig. 2). Patients received a median of 4 LeMLAR cycles (range 1–6; median cycle duration, 28 days, range 27–69; median time on lenalidomide, 21 days per cycle, range 7–22; treatment delays, 22 of a total of 76 cycles [29%]; outpatient administration, 69 cycles [91%]). Eight of 12 patients reaching cycle 3 were escalated to chemotherapy level 3, and six of nine patients reaching cycle 5 were escalated to level 4. In all but one case, escalated doses were maintained or further increased in subsequent cycles.

By immunohistochemistry, ten each of 20 evaluable phases I/II biopsies were allocated to the germinal or non-germinal center B cell subtype, respectively^9^. Statistically significant differences in ORR (70% versus 40%, Fisher’s exact test *p* = 0.370), PFS (7.1 versus 3.2 months, log-rank test *p* = 0.76), or OS (19.6 versus 7.7 months, log-rank test *p* = 0.092) were not observed (Supplementary Fig. 3). One of 18 biopsies evaluable by fluorescence-in-situ-hybridization showed a double-hit lymphoma (PD).

There was one treatment-related grade 5 septicemia in phase I (25 mg lenalidomide, chemotherapy level 2) and one disease-related grade 5 ileus in phase II (Supplementary Table 3). Grade 3-4 adverse events occurring in >1 patient in phase I/II included anemia (23%/25%), infection (23%/15%), thrombocytopenia (18%/20%), neutropenia (9%/20%), leukopenia (9%/5%), and diarrhea (14%/5%). Grade 1–2 adverse events occurring in >2 patients comprised creatinine increase (41%/40%), fever (27%/45%), infection (14%/30%), nausea (27%/15%), constipation (23%/10%), diarrhea (14%/15%), vomiting (14%/15%), fatigue (5%/25%), rash (9%/15%), and neutropenia (5%/15%). Alopecia was not observed. G-CSF was used in 30 cycles (39.5%), with increasing frequency from cycle 1 (25%) to cycle 6 (75%). Antibiotics were given in 17 cycles (22.4%), for a median duration of 7.5 days (range, 3–15). Red blood cell or platelet transfusions were given in 11 (14.5%) and 4 cycles (5.3%), respectively.

The behavior of blood lymphocytes (essentially representing natural killer cells and T cells in rituximab-treated patients) varied. Rising counts in the first two cycles were correlated with CR (four CR among six patients with rising counts and evaluable treatment response), stable counts with PR (3/6), and declining counts with SD/PD (5/6; Fisher’s exact test *p* = 0.046). Complete responders tended to maintain counts above baseline throughout treatment (Supplementary Table 4). The correlation between lymphocyte response and tumor response was independent of absolute cell numbers, which, in complete responders, ranged from 0.23 /nl to 3.07 /nl at baseline (normal range, 1.0–3.4).

LeMLAR was well tolerated. The most common adverse event was an infection, occurring in almost half the patients. Antimicrobial prophylaxis was not mandatory, and G-CSF was only given to raise neutrophils from <0.5 /nl to >1.0 /nl. Systematic antimicrobial prophylaxis and more extensive G-CSF use may further improve the protocol’s tolerability. ORR (55%) was high. Lymphocytes tended to rise in complete responders and fall in non-responders. The reason for this observation remained unresolved. Median DOR to LeMLAR was twice as long as DOR to the previous line of therapy (19.4 versus 9.5 months). These results compare favorably with r/rDLBCL treated with lenalidomide alone (ORR 26%; DOR 6 months)^1^ or in combination with rituximab (ORR 33%; DOR 10.2 months)^1^ or ibrutinib and rituximab (ORR 38%; DOR 15.9 months)^10^. Our results resemble those recently reported for lenalidomide and tafasitamab (ORR 60%; DOR 21.7 months)^11^. It is tempting to speculate that methotrexate and cytarabine may further enhance this regimen’s efficacy.

Previous lenalidomide-chemotherapy combinations were restricted to first-line and second-line therapies^12^, precluding comparison with LeMLAR. In a recent review, the efficacy of salvage regimens not including lenalidomide varied widely, depending on patient selection and intervention (median ORR 44.9%, range 2–83; DOR 9.5 months, range 3.0–17.3)^13^. Polatuzumab vedotin, bendamustine, and rituximab, recently approved for r/rDLBCL, yielded an ORR of 45.0%, with a DOR of 12.6 months^14^. Chimeric antigen receptor (CAR) T cells produced response rates of 52–83% in patients able to receive the bioengineered product (comprising 67–91% of recruited patients), with a median DOR of 11.1 months to not reached^15^. Similar to other studies^13,14^, grade ≥3 adverse events were frequent^15^. Taken together, LeMLAR achieved disease control similar to other novel treatments for r/rDLBCL. The safety profile appears more favorable.

## Supplementary information

LeMLAR Supplementary Information

## References

[1] Witzig TE (2015). A comprehensive review of lenalidomide therapy for B-cell non-Hodgkin lymphoma. Ann. Oncol..

[2] Gaynor ER, Ultmann JE, Golomb HM, Sweet DL (1985). Treatment of diffuse histiocytic lymphoma (DHL) with COMLA (cyclophosphamide, oncovin, methotrexate, leucovorin, cytosine arabinoside): a 10-year experience in a single institution. J. Clin. Oncol..

[3] Gribben JG, Fowler N, Morschhauser F (2015). Mechanisms of action of lenalidomide in B-cell non-Hodgkin lymphoma. J. Clin. Oncol..

[4] Matheson DS, Green B, Hoar DI (1983). The influence of methotrexate and thymidine on the human natural killer cell function in vitro. J. Immunol..

[5] Markasz L (2007). Effect of frequently used chemotherapeutic drugs on the cytotoxic activity of human natural killer cells. Mol. Cancer Ther..

[6] Ersvaer, E., Brenner, A. K., Vetås, K., Reikvam, H. & Bruserud, Ø. Effects of cytarabine on activation of human T cells - cytarabine has concentration-dependent effects that are modulated both by valproic acid and all-trans retinoic acid. *BMC Pharmacol. Toxicol.* **16**, 12 (2015). 10.1186/s40360-015-0012-2 PMC4422044 25934555

[7] Wiernik PH (2008). Lenalidomide monotherapy in relapsed or refractory aggressive non-Hodgkin’s lymphoma. J. Clin. Oncol..

[8] Cheson BD (1999). Report of an international workshop to standardize response criteria for non-Hodgkin’s lymphomas. NCI Sponsored International Working Group. J. Clin. Oncol..

[9] Hans CP (2004). Confirmation of the molecular classification of diffuse large B-cell lymphoma by immunohistochemistry using a tissue microarray. Blood.

[10] Goy A (2019). Ibrutinib plus lenalidomide and rituximab has promising activity in relapsed/refractory non-germinal center B-cell-like DLBCL. Blood.

[11] Salles G (2020). Tafasitamab plus lenalidomide in relapsed or refractory diffuse large B-cell lymphoma (L-MIND): a multicentre, prospective, single-arm, phase 2 study. Lancet Oncol..

[12] Kühnl A (2020). R-GEM-Lenalidomide versus R-GEM-P as second-line treatment of diffuse large B-cell lymphoma: results of the UK NRCI phase II randomised LEGEND trial. Ann. Hematol..

[13] Salles GA (2019). Treatment of aggressive B-cell non-Hodgkin lymphoma beyond frontline therapy in patients not eligible for stem cell transplantation: a structured review. Leuk. Lymphoma.

[14] Sehn LH (2020). Polatuzumab vedotin in relapsed or refractory diffuse large B-cell lymphoma. J. Clin. Oncol..

[15] Abramson JS, Lunning M, Palomba ML (2019). Chimeric antigen receptor T-cell therapies for aggressive B-cell lymphomas: current and future state of the art. Am. Soc. Clin. Oncol. Educ. Book.

