# Supplementary material for: Phase I/II trial of lenalidomide, methotrexate, leucovorin, cytarabine, and rituximab (LeMLAR) in relapsed or refractory diffuse large B cell lymphoma
Source: Blood Cancer J. 2021 May 17;11(5):95. doi: 10.1038/s41408-021-00485-5 (PMC8129096; doi:10.1038/s41408-021-00485-5)
Supplement: Supplementary file 1 — LeMLAR Supplementary Information [file 41408_2021_485_MOESM1_ESM.pdf]

Supplementary Information

Phase I/II trial of lenalidomide, methotrexate, leucovorin, cytarabine, and rituximab (LeMLAR) in relapsed or refractory diffuse large B-cell lymphoma

Ulrich Dührsen, Mareike Tometten, Frank Kroschinsky, Arnold Ganser, Stefan Ibach, Stefanie Bertram, Andreas Hüttmann

Supplementary Tables

1. Previous therapies
2. Response to LeMLAR in relation to response to last treatment in phase II patients
3. Adverse events
4. Blood lymphocyte counts in relation to treatment response in phase II patients

Supplementary Figures

1. Response duration in phase II patients
2. Progression-free survival and overall survival in phase II patients
3. Progression-free survival and overall survival in germinal and non-germinal center B-cell lymphomas

LeMLAR Trial Protocol, Version 1.07

Supplementary Table 1. Previous therapies.

**Line of therapy**[a]	**Phase I** (n=22)	**Phase II** (n=20)
**First line**		
R-CHOP	21[b]	19
Rituximab-bendamustine	1	0
Radiotherapy	0	1
**Second line**		
Autologous transplantation	4	6
Allogeneic transplantation	0	1
R-DHAP	6	2
R-GDP	0	1
R-GemOx	2	3
R-ICE	0	1
R-CHOP	2	2
Rituximab-bendamustine	1	0
Radiotherapy	0	1
**Third line**		
Allogeneic transplantation	1	0
R-DHAP	1	0
R-GemOx	1	0
R-ICE	3	1
MTX-based Burkitt's lymphoma protocol	1	0
Rituximab-pixantrone	0	1
Rituximab-bendamustine	1	1
Tafasitamab-bendamustine	0	1
Radiotherapy	2	2
**Fourth line**		
R-DHAP	1	0
DexaBEAM	1	1
Radiotherapy	1	0

[a]Radiotherapy was counted as an independent line of therapy only, if it was used as a stand-alone measure temporally unrelated to chemotherapy; [b]number of patients.

R-CHOP, rituximab, cyclophosphamide, doxorubicin, vincristine, prednisone; R-DHAP, rituximab, dexamethasone, cytarabine, cisplatin; R-GDP, rituximab, gemcitabine, dexamethasone, cisplatin; R-GemOx, rituximab, gemcitabine, oxaliplatin; R-ICE, rituximab, ifosfamide, carboplatin, etoposide; DexaBEAM, dexamethasone, carmustine, etoposide, cytarabine, melphalan. Note that eight patients from phase I were also included in phase II.

Supplementary Table 2. Response to LeMLAR in relation to response to last treatment in phase II patients.

Patient identifier	Lines of prior therapy	Last treatment		
		Regimen	Response	Response duration
**LeMLAR - Complete remission (CR)**				
401-09	1	R-CHOP	CR	9 months
001-04	2	Allogeneic transplantation	CR	6 months
001-03	2	Autologous transplantation	PR	13 months
419-09	3	Tafasitamab-bendamustine	PR	1 month
401-20	2	Autologous transplantation	SD	5 months
**LeMLAR - Partial remission (PR)**				
401-14	3	Rituximab-pixantrone	CR	32 months
401-23	2	Autologous transplantation	CR	19 months
401-18	2	Autologous transplantation	CR	10 months
401-07	3	Rituximab-bendamustine	PD	n.a.
401-08	1	R-CHOP	PD	n.a.
401-21	3	Radiotherapy[a]	PD	n.a.
**LeMLAR - Stable disease (SD)**				
401-17	2	R-GDP	PD	n.a.
419-05	2	R-DHAP	PD	n.a.
419-04	4	DexaBEAM	PD	n.a.
**LeMLAR - Progressive disease (PD)**				
401-16	1	R-CHOP	PD	n.a.
401-15	2	R-GemOx	PD	n.a.
401-22	2	Radiotherapy	PD	n.a.
419-03	2	R-DHAP	PD	n.a.
**LeMLAR - Not evaluable**				
401-10	2	R-GemOx	PD	n.a.
001-01	3	Radiotherapy[a]	PD	n.a.

[a]Previous second-line autologous transplantation associated with progressive disease.

See footnote to Supplementary Table 1 for composition of chemotherapy protocols; n.a., not applicable.

Supplementary Table 3. Adverse events.

	**Phase I** (n=22)				**Phase II** (n=20)			
**Adverse event / grade[a]**	**1**	**2**	**3**	**4**	**1**	**2**	**3**	**4**
Anemia	0	1[b]	6	0	0	2	5	0
Leukopenia	0	0	1	1	0	0	1	0
Neutropenia	1	0	2	0	2	1	4	0
Thrombocytopenia	0	1	4	0	0	1	2	2
Fever	5	1	1	0	6	3	0	0
Infection[c]	0	3	5	0	3	3	2	1
Pruritus	1	0	0	0	2	0	0	0
Rash	0	2	0	0	0	3	1	0
Fatigue	0	1	0	0	4	1	1	0
Nausea	4	2	0	0	2	1	0	0
Vomiting	2	1	1	0	2	1	1	0
Anorexia	0	0	0	0	0	1	0	0
Dysgeusia	0	1	0	0	1	0	0	0
Conjunctivitis	0	0	0	0	0	1	0	0
Oral mucositis	0	1	1	0	0	2	0	0
Gastritis	0	1	0	0	0	2	0	0
Diarrhea	1	2	3	0	3	0	1	0
Constipation[d]	3	2	0	0	2	0	0	0
Bilirubine increase	0	0	1	0	0	0	1	0
ALT increase	0	0	1	0	0	0	0	0
Creatinine increase	7	2	1	0	7	1	0	0
Hypercalcemia	0	0	1	0	0	1	0	0
Sensory neuropathy	1	0	0	0	1	0	0	0
Hemorrhage	2	0	0	0	0	1	0	0
Thromboembolism	0	0	1	0	0	1	0	0

[a]Highest grade observed per patient; [b]number of patients; [c]in addition, one treatment-related grade 5 septicemia in phase I; [d]in addition, one lymphoma-related grade 5 ileus in phase II.

ALT, alanine aminotransferase. Note that eight patients from phase I were also included in phase II.

Supplementary Table 4. Blood lymphocyte counts in relation to treatment response in phase II patients.

	**Complete remission** (n=6)	**No complete remission** (n=12)	
	Median (range)	Median (range)	**p**
Baseline	0.83 /nl (0.23 - 3.07)	1.17 /nl (0.4 - 2.32)	0.385
Cycle 1	144 % (76 - 315)	66 % (19 - 478)	< 0.001
Cycle 2	143 % (32 - 191)	78 % (8 - 285)	0.001
Cycle 3	113 % (73 - 155)	58 % (23 - 144)	< 0.001
Cycle 4	116 % (58 - 191)	57 % (6 - 114)	< 0.001
Cycle 5	101 % (73 - 157)	48 % (11 - 217)	< 0.001
Cycle 6	129 % (61 - 222)	68 % (20 - 201)	0.007

Lymphocytes were measured on days 1, 8, 15, and 22 of each 28-day cycle; normal range at baseline (cycle1, day 1), 1.0-3.4 /nl; cycles 1-6, lymphocyte count in relation to baseline; p, Mann-Whitney U test.

Supplementary Figure 1

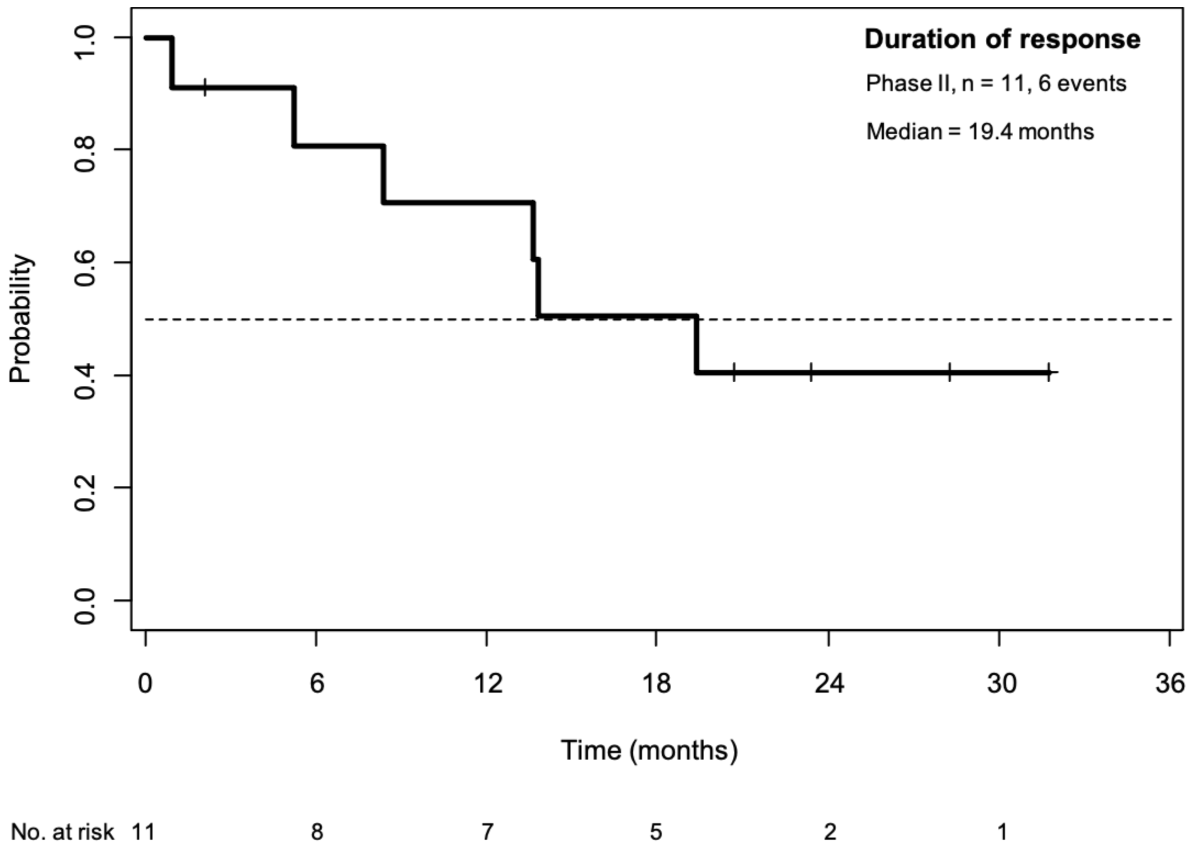

Response duration in phase II patients treated at the maximum tolerated dose level

Supplementary Figure 2

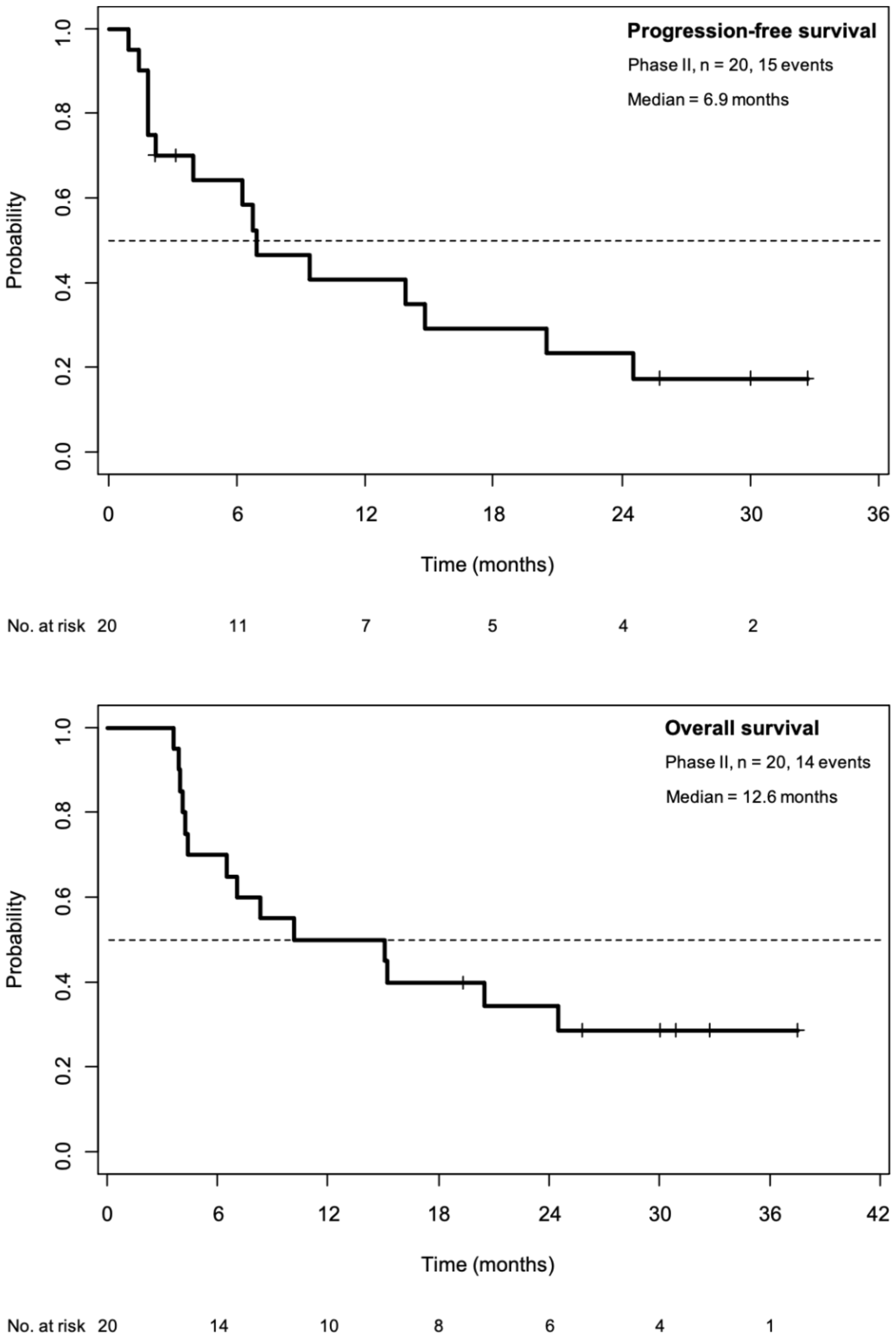

Progression-free survival (top) and overall survival (bottom) in phase II patients treated at the maximum tolerated dose level

Supplementary Figure 3

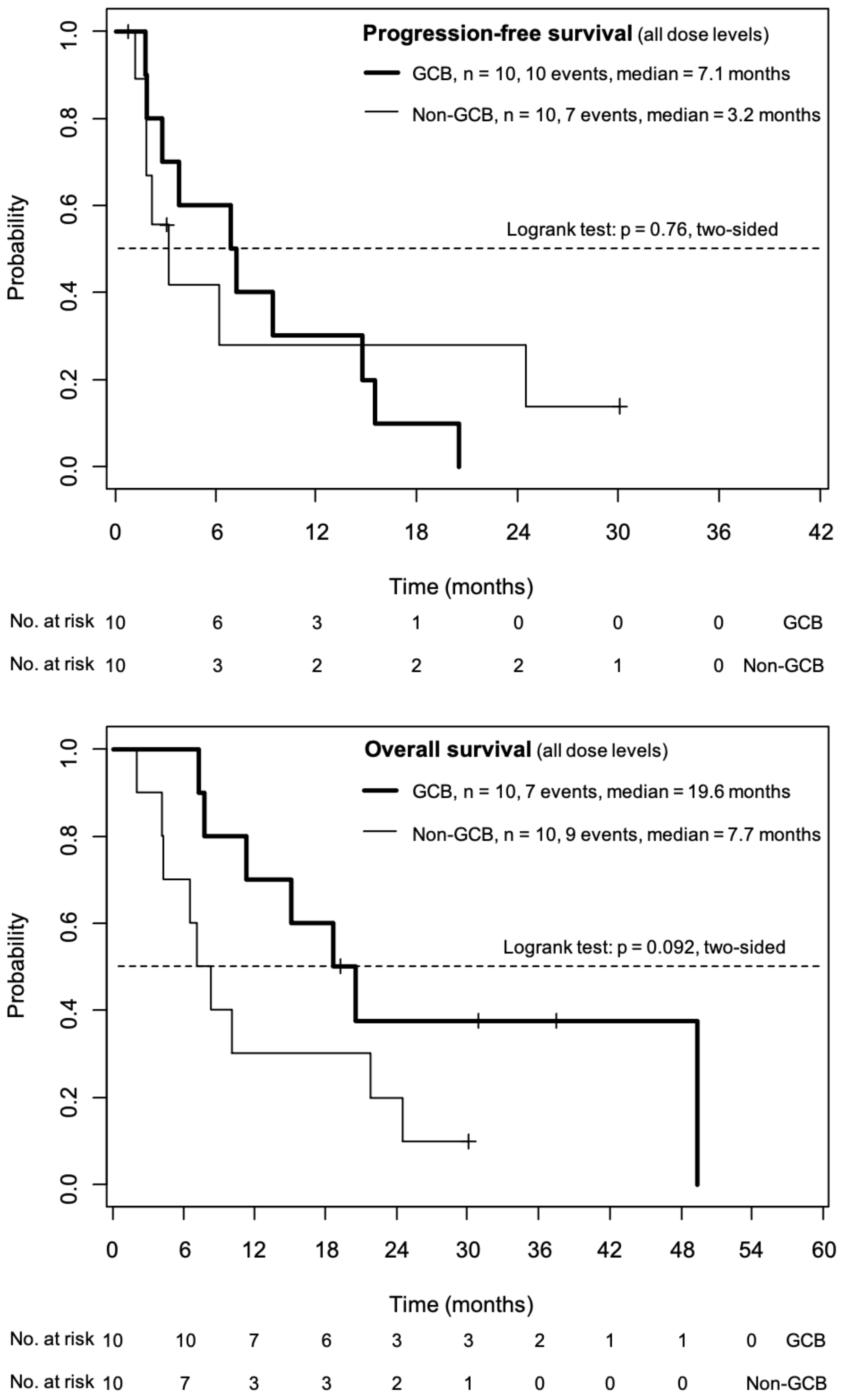

Progression-free survival (top) and overall survival (bottom) in germinal center (GCB) and non-germinal center B-cell lymphomas (non-GCB) (all dose levels)

Lenalidomide in conjunction with methotrexate, leucovorin, cytarabine and rituximab for the treatment of relapsed or refractory CD20-positive aggressive lymphomas: an open-label, multicenter phase I/II trial (the LeMLAR trial)

**Investigational product (IP):**	**Lenalidomide**
**Trial number:**	**03-2012**
**Protocol code:**	**LeMLAR**
**Protocol number and date:**	**Version 1.07, July 1, 2014**
**EudraCT number:**	**2012-001891-13**
**ClinicalTrials.gov identifier:**	**NCT01788189**

CONFIDENTIAL

This protocol is provided to you as an investigator, potential investigator, or consultant for review by you, your staff, and ethics committee/institutional review board. The information contained in this document is regarded as confidential and, except to the extent necessary to obtain informed consent, may not be disclosed to another party unless such disclosure is required by law or regulations. Persons to whom the information is disclosed must be informed that the information is confidential and may not be further disclosed by them.

SPONSOR, COORDINATORS AND BIOMETRICIANS

Sponsor according to German drug law (Arzneimittelgesetz)

University Hospital Essen
represented by the Administrative Director Hans-Peter Tappe
Hufelandstraße 55
45122 Essen
Germany
Telefone: 0201-723-2602
Telefax: 0201-723-5921
E-mail: hanspeter.tappe@uk-essen.de

Coordinating Principal Investigator

Prof. Dr. Ulrich Dührsen
Department of Hematology
University Hospital Essen
University of Duisburg-Essen
Hufelandstraße 55
45122 Essen
Germany
Telefone: 0201-723-2417
Telefax: 0201-723-5928
E-mail: ulrich.duehrsen@uk-essen.de

Trial Coordinator and Trial Office

Priv.-Doz. Dr. Andreas Hüttmann
PETAL Trial Office / Department of Hematology
University Hospital Essen
University of Duisburg-Essen
Hufelandstraße 55
45122 Essen
Germany
Telefone: 0201-723-1861
Telefax: 0201-723-5189
E-mail: petal@uk-essen.de

Biometrical Planning

Prof. Dr. Karl-Heinz Jöckel
Clinical Trials Center Essen (ZKSE)
University Hospital Essen
University of Duisburg-Essen
Hufelandstraße 55
45122 Essen
Germany
Telefone: 0201-723-4513
Telefax: 0201-723-5933
E-mail: k-h.joeckel@uk-essen.de

Dr. André Scherag
Clinical Trials Center Essen (ZKSE)
University Hospital Essen
University of Duisburg-Essen
Hufelandstraße 55
45122 Essen
Germany
Telefone: 0201-723-4793
Telefax: 0201-723-5933
E-mail: andre.scherag@uk-essen.de

SPONSOR SIGNATURE PAGE

By signing below, the sponsor agrees to adhere to the protocol as outlined and agrees that any changes to the protocol must be approved by the director of the clinical investigation, prior to seeking approval from the Ethics Committee. This study will be conducted in accordance with current International Conference on Harmonisation (ICH) guidelines for Good Clinical Practice (GCP), the Declaration of Helsinki, and local ethical and legal requirements.

____________	____
Date	Signature of Sponsor – University Hospital Essen<br>(represented by the Administrative Director Hans-Peter Tappe)

COORDINATING PRINCIPAL INVESTIGATOR SIGNATURE PAGE

By my signature, I agree the protocol has been written to comply with ICH Good Clinical Practices guidelines and agree to offer guidance throughout the study as needed.

____________	____
Date	Signature of Coordinating Principal Investigator – Prof. Dr. Ulrich Dührsen

SITE PRINCIPAL INVESTIGATOR SIGNATURE PAGE

Printed Name of Site Principal Investigator and Title

Site Number:________

Institution Name:_______________________________________

By my signature, I agree to personally supervise the conduct of this study at my study site and to ensure its conduct is in compliance with the protocol, informed consent, Institutional Review Board / Ethics Committee procedures, the Declaration of Helsinki, ICH Good Clinical Practices Guidelines, and local regulations governing the conduct of clinical studies.

___________ ____

Date Signature of Site Principal Investigator

PROTOCOL SUMMARY

Full Study Title	Lenalidomide in conjunction with methotrexate, leucovorin, cytarabine and rituximab for the treatment of relapsed or refractory CD20-positive aggressive lymphomas: an open-label, multicenter phase I/II trial
Short Study Title	LeMLAR trial
Indication	Relapsed or refractory CD20-positive aggressive B-cell lymphomas
Primary Objectives	<u>Phase I:</u><br>Evaluation of the feasibility, safety and dose-limiting toxicity of the LeMLAR regimen<br><u>Phase II:</u><br>Evaluation of the efficacy of the LeMLAR regimen at the maximum tolerated dose
Secondary Objectives	Evaluation of the types of treatment response, their durability, the pattern of relapse, long-term toxicities, secondary malignancies, overall survival
Study Design	Multicenter prospective open-label non-randomised phase I/II study<br><u>Phase I:</u><br>Dose escalation of methotrexate and cytarabine in a 3 + 3 design with fixed doses of lenalidomide and rituximab<br><u>Phase II:</u><br>Treatment of 20 patients at maximum tolerated doses of phase I
Study Population	Patients above the age of 18 years with relapsed or refractory CD20-positive aggressive B-cell lymphomas with an ECOG performance status of 0 – 3 and no significant comorbidities
Study Treatments	<u>The LeMLAR protocol:</u><br><u>Le</u>nalidomide 25 mg p.o., days 1 – 21<br><u>M</u>ethotrexate 30 – 60 – 90 – 120 – 150 mg/m² i.v. bolus, days 1, 8, 15<br><u>L</u>eucovorin 4 x 45 mg p.o. (every 6 hrs), days 2, 9, 16<br>Cytarabine (<u>A</u>ra-C) 75 – 150 – 225 – 300 – 375 mg/m² i.v. bolus, days 1, 8, 15<br><u>R</u>ituximab 375 mg/m² i.v. infusion, day 1<br><br>28-day cycles, maximum 6 cycles, definition of dose-limiting toxicity in cycles 1 and 2, intra-patient dose escalation after cycles 2 and 4 in case of absence of dose-limiting toxicity in previous cycles<br><br>The dose level below the level where dose-limiting toxicity is observed defines the maximum tolerated doses of methotrexate and cytarabine. However, if, at a lenalidomide dose of 25 mg, methotrexate and cytarabine cannot be escalated beyond the first dose level, the lenalidomide dose will be reduced to 20 mg. If, at 20

	mg, methotrexate and cytarabine cannot be escalated beyond the first dose level, the lenalidomide dose will be reduced to 15 mg. If, at 15 mg, methotrexate and cytarabine cannot be escalated beyond the first dose level, dose-limiting toxicity is reached and the maximum tolerated dose is the highest dose of lenalidomide able to be combined with the first dose level of methotrexate and cytarabine in a 3 + 3 phase I design.
Primary Endpoints	<u>Phase I:</u><br>Dose-limiting toxicity of the combination regimen, determination of the maximum tolerated doses of methotrexate and cytarabine<br><br><u>Dose-limiting toxicities</u>:<br>- any of the following on the day of methotrexate/cytarabine injection (day 8 + ≤ 3 days / day 15 + ≤ 6 days of the first or second treatment cycle; day 1 of the second or third cycle which is equivalent to day 29 + ≤ 7 days of the previous cycle):<br>- neutrophils < 500/µl<br>- platelets < 25.000/µl<br>- creatinine clearance < 60 ml/min<br>- bilirubin ≥ 3,0 mg/dl<br>- serum AST/GOT or ALT/GPT ≥ 6 x upper limit of normal<br>- mucositis grade 3 or 4<br><br>- requirement for dose reduction of methotrexate/cytarabine in the first or second treatment cycle<br>- fewer than 21 days of lenalidomide in the first or second treatment cycle<br>- toxicity-related delay of second or third treatment cycle by more than 7 days<br>- any other toxicity preventing continuation of therapy according to protocol in the first or second treatment cycle (except allergic reactions)<br><br><u>Phase II:</u><br>Overall response rate (percentage of complete and partial remissions combined)
Secondary Endpoints	Rates of stable disease and progressive disease, relapse rate, progression-free survival, event-free survival, disease-free survival, overall survival, toxicity (type, onset, duration), secondary malignancies
Expected Study Time Schedule	Activation: January 1, 2013<br>Recruitment: January 1, 2013 – December 31, 2015<br>Last patient treatment: December 31, 2015 – June 30, 2016<br>Last patient follow-up: July 1, 2016 – June 30, 2017<br>Analysis: July 1, 2017 – December 31, 2017<br>Final report: December 31, 2017<br><br>To assess survival parameters, long-term toxicities and secondary malignancies follow-up will be extended outside the funding period of the study until the patients' death in the setting of regular patient care.

FLOWCHART

The LeMLAR Protocol

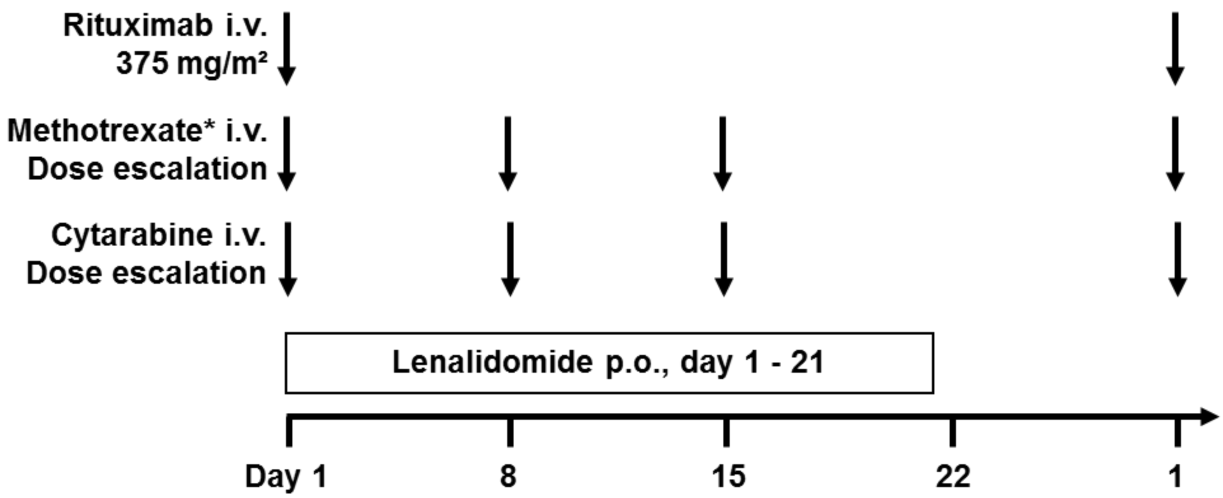

* with oral leucovorin rescue

The LeMLAR Trial

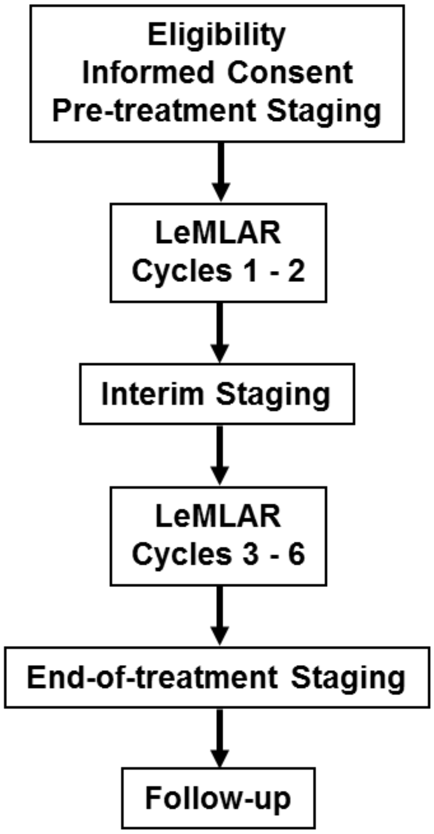

TABLE OF CONTENTS

Chapter		Page
TITLE PAGE		1
SPONSOR, COORDINATORS AND BIOMETRICIANS		2
SPONSOR SIGNATURE PAGE		3
COORDINATING PRINCIPAL INVESTIGATOR SIGNATURE PAGE		4
SITE PRINCIPAL INVESTIGATOR SIGNATURE PAGE		5
PROTOCOL SUMMARY		6
FLOWCHART		8
TABLE OF CONTENTS		9
1.	INTRODUCTION	13
2.	STUDY OBJECTIVES	14
2.1.	Primary Objectives	14
2.2.	Secondary Objectives	14
3.	STUDY ENDPOINTS	14
3.1.	Primary Endpoints	14
3.2.	Secondary Endpoints	14
4.	OVERALL STUDY DESIGN	15
4.1.	Study Design	15
4.2.	Study Design Rationale	15
5.	STUDY DURATION	16
6.	STUDY POPULATION	16
6.1.	Number of Subjects and Sites	16
6.2.	Inclusion Criteria	16
6.3.	Exclusion Criteria	17
7.	IDENTIFICATION AND REGISTRATION OF PATIENTS	17

8. PROCEDURES 18
8.1. Pre-Treatment Staging 18
8.2. Investigations in the Course of Treatment 19
8.3. Interim Staging (after the first two treatment cycles) 19
8.4. End-of-Treatment Staging (4 weeks after the last treatment cycle) 19
8.5. Follow-up 20
9. DESCRIPTION OF STUDY TREATMENTS 20
9.1. Description of Investigational Products 20
9.1.1. Cytarabine (e.g. ARA-cell®) 20
9.1.2. Lenalidomide (Revlimid®) 21
9.1.3. Methotrexate (e.g. Methotrexat medac) and Folinic Acid (Leucovorin®) 22
9.1.4. Rituximab (MabThera®) 22
9.2. Treatment Administration and Schedule 23
9.3. Treatment Assignment in Phase I and Phase II 24
9.4. Temporary Discontinuation of Treatment and Dose Reduction 27
9.5. Packaging, Labeling, Accountability and Disposal of Lenalidomide 27
10. STATISTICAL ANALYSES 28
10.1. Study Endpoints Definition 28
10.2. Study Population Definitions 28
10.3. Sample Size and Power Considerations 29
10.4. Background and Demographic Characteristics 29
10.5. Subject Disposition 29
10.6. Efficacy Analysis 29
10.7. Safety Analysis 30
10.8. Interim Analysis 30

11. ADVERSE EVENTS 30
11.1. Monitoring, Recording and Reporting of Adverse Events 30
11.2. Evaluation of Adverse Events 31
11.2.1. Seriousness 31
11.2.2. Severity 32
11.2.3. Causality 32
11.2.4. Duration 32
11.2.5. Action Taken 33
11.2.6. Outcome 33
11.3. Abnormal Laboratory Values 33
11.4. Pregnancy 33
11.4.1. Pregnancy Prevention Program 33
11.4.2. Reporting Procedures for Pregnancies 33
11.5. Immediate Reporting of Serious Adverse Events 34
11.5.1. Safety Queries 35
11.6. Expedited Reporting of Adverse Events (SUSARs) 35
12. DISCONTINUATIONS AND TREATMENT AFTER THE END OF TRIAL PARTICIPATION 35
12.1. Discontinuations 35
12.2. Treatment after the end of trial participation 35
13. REGULATORY CONSIDERATIONS 36
13.1. Good Clinical Practice 36
13.2. Investigator Responsibilities 36
13.3. Subject Information and Informed Consent 36
13.4. Confidentiality 37
13.5. Protocol Amendments 37

13.6. Ethics Committee / Regulatory Authorities Review and Approval 37
13.7. Closure of the Study 37
13.8. Study Subject Insurance 38
13.9. Financial Aspects 38
14. DATA HANDLING AND RECORDKEEPING 38
14.1. Data/Documents 38
14.2. Data Management 38
14.3. Record Retention 39
15. QUALITY CONTROL AND QUALITY ASSURANCE 39
15.1. Study Monitoring and Source Data Verification 39
15.2. Audits and Inspections 40
16. PUBLICATIONS 40
17. REFERENCES 40
18. APPENDICES
18.1. Appendix 1: Table of Events
18.2. Appendix 2: Ann Arbor Staging System
18.3. Appendix 3: Performance Status (ECOG)
18.4. Appendix 4: International Prognostic Index (IPI)
18.5. Appendix 5: Informed Consent Form (in German)
18.6. Appendix 6: Lenalidomide Pregnancy Prevention Risk Management Plan
18.7. Appendix 7: Study Subject Insurance Policy (in German)
18.8. Appendix 8: Subject Identification Code List
18.9. Appendix 9: Product Information Sheets (in German)
18.10 Appendix 10 Delegation of Responsibility (in German)
18.11 Appendix 11 Agreement with Participating Trial Centers (in German)

1. INTRODUCTION

Approximately 40% of patients with aggressive B-cell lymphoma will relapse following initial immunochemotherapy (1-5). Although a significant number of patients with relapsed disease can be salvaged with high-dose chemotherapy, the majority will succumb to the disease (6). Thus, the development of more effective treatments is essential to improve long-term outcome in aggressive B-cell lymphoma.

Lenalidomide is an antineoplastic agent with pleiotropic actions targeting the tumor cells, the immune system and the microenvironment (7). When given until disease progression or intolerance at a daily dose of 25 mg in a 3-weeks-on/1-week-off schedule lenalidomide showed considerable single agent activity in relapsed or refractory aggressive B cell lymphomas in two consecutive phase II trials, with complete remission rates of 12% or 13%, respectively, and an overall response rate of 35% (8,9). Response rates were somewhat lower in diffuse large B-cell lymphoma, the most common type of aggressive B-cell lymphoma (overall response rate: 19% or 28%; complete remission rate: 12% or 7%). Interestingly, responses were more frequent in the non-germinal center B-cell type of diffuse large B-cell lymphoma (53%) than in the germinal center B-cell type (9%) (10).

The monoclonal CD20 antibody rituximab has improved cure rates in aggressive B cell lymphomas by 10 % to 15 % (1-5). It is considered an indispensable constituent of any treatment for CD20-positive aggressive lymphomas. However, combining a submaximal dose of lenalidomide (20 mg, day 1 – 21) with rituximab (375 mg/m², days 1 and 21) in four 4-week cycles followed by lenalidomide maintenance in elderly patients with relapsed or refractory diffuse large B-cell lymphoma failed to produce a response rate greater than 40% which was considered a prerequisite for testing the two-drug combination further (11). To reach this goal it appears promising to combine lenalidomide and rituximab with classic cytotoxic drugs with documented activity in B-cell lymphomas. In first-line therapy combination of lenalidomide with the R-CHOP regimen proved feasible in a recent phase I trial (12).

In order to exploit the full antineoplastic potential of lenalidomide in combination therapy, it should be given at a dose and schedule that has previously been shown to be effective as a monotherapy. The toxicity of the combination partners should not overlap with that of lenalidomide which is characterized by moderate hematotoxicity (grade 3 or 4 leukopenia: 8% - 14%; thrombocytopenia: 19% - 20%) (8,9). The basic principle of lenalidomide monotherapy, i.e. continuous exposure of the tumor to the antineoplastic agent, should also be reflected in the administration schedule of the other cytotoxic drugs.

Several other aspects are of importance in the treatment of patients with relapsed or refractory lymphomas not qualifying for curative high-dose therapy. First, the cytotoxic agents employed should differ from those used in previous treatment phases. Second, long-term toxicities from previous treatments, such as neuropathy induced by vinca alkaloids or cisplatin, renal damage induced by cisplatin or ifosfamide or cardiac damage induced by anthracyclines, should not be worsened by salvage therapy. Third, severe neutropenia by highly myelotoxic agents and hyperglycemia by steroids should be avoided to enable treatment in an outpatient setting.

The above requirements are likely to be met by combining lenalidomide in a 3-weeks-on/1-week-off schedule with weekly methotrexate and cytarabine and four-weekly rituximab. In the 1970s and 1980s weekly bolus injections of methotrexate and cytarabine were shown to be highly effective in the treatment of aggressive lymphomas. In the COMLA regimen, methotrexate at a dose of 120 mg/m² (with oral leucovorin rescue) and cytarabine at a dose of 300 mg/m² were administered once weekly for 8 consecutive weeks (13,14). Each 8-week methotrexate/cytarabine treatment cycle was preceded by a single dose of the myelotoxic drug cyclophosphamide (1,5 g/m²) and 3 weekly doses of vincristine (1,4 mg/m²) and followed by a 2-week rest period. In patients with aggressive lymphomas, the COMLA regimen yielded treatment

results similar to those obtained with the CHOP regimen (14). It was dropped in the 1980s because “third generation” protocols such as ProMACE-MOPP, COP-BLAM, MACOP-B or M-BACOD were deemed more effective than CHOP or COMLA, an assumption that, for the CHOP regimen, was shown to be wrong in a randomized trial (15).

The present study aims at improving the efficacy of lenalidomide and rituximab by adding methotrexate and cytarabine. To highlight its derivation from the COMLA regimen the novel treatment protocol will be termed ‘LeMLAR’. Lenalidomide will be given at a fixed daily dose of 25 mg for the first three weeks of each four-week treatment cycle for a maximum of six cycles. On day 1 of each cycle, rituximab will be infused at a dose of 375 mg/m². On days 1, 8 and 15, methotrexate (with oral leucovorin rescue) and cytarabine will be given as intravenous bolus injections. In the phase I part of the study dose limiting toxicity (DLT) of the regimen will be determined by escalating the doses of methotrexate and cytarabine in a 3 + 3 design. If methotrexate and cytarabine cannot be escalated beyond the first dose level, the lenalidomide dose will be reduced to 20 mg with possible further reduction to 15 mg (cf. sections 9.2 and 9.3). In the phase II part response rates, toxicities and survival times of patients treated at the maximum tolerated dose (MTD) will be evaluated.

If the LeMLAR regimen can be shown to induce long-term remissions in a substantial proportion of patients, it may constitute an appropriate backbone for the design of first-line strategies in non-fit patients with aggressive B-cell lymphomas. Owing to a changing demography, this group of patients is continuously increasing, and no satisfactory treatment options are available at the present time. Advantages of the LeMLAR regimen in elderly patients with aggressive lymphomas include the availability of its major constituent as a tablet, its steroid-free composition, its expected good tolerability and a once weekly physician contact throughout the treatment period.

2. STUDY OBJECTIVES

2.1. Primary Objective

The primary objective of the phase I part of the study is to assess the feasibility, safety and dose-limiting toxicity of the LeMLAR regimen. The primary objective of the phase II part is to determine its efficacy at the maximum tolerated dose.

2.2. Secondary Objectives

Secondary objectives of the study are to evaluate the types of treatment response, their durability, the pattern of relapse, long-term toxicities, second primary malignancies and overall survival.

3. STUDY ENDPOINTS

3.1. Primary Endpoints

Phase I: - dose-limiting toxicity of the combination regimen
- maximum tolerated doses of methotrexate and cytarabine

Phase II: - overall response rate (percentage of complete and partial remissions combined)

3.2. Secondary Endpoints

Rates of stable disease and progressive disease, relapse rate, progression-free survival,

event-free survival, disease-free survival, overall survival, toxicity (type, onset, duration), secondary malignancies

4. OVERALL STUDY DESIGN

4.1. Study Design

The LeMLAR trial is a prospective open-label non-randomised multicenter phase I / II study in patients with relapsed or refractory CD20-positive aggressive B-cell lymphomas.

Phase I
Lenalidomide will be given at a fixed daily dose of 25 mg for the first three weeks of each four-week treatment cycle for a maximum of six cycles. On day 1 of each cycle, rituximab will be infused at a dose of 375 mg/m². On days 1, 8 and 15, methotrexate (with oral leucovorin rescue) and cytarabine will be given in escalating doses as intravenous bolus injections.

Dose limiting toxicity will be determined by increasing the doses of methotrexate and cytarabine in sequential patient cohorts in a 3 + 3 design. Because tolerance to lenalidomide and cytotoxic agents are likely to decrease with increasing numbers of treatment cycles in pretreated patients with aggressive lymphomas, evaluation of dose-limiting toxicity will be confined to the first two cycles. The requirement for dose reductions in subsequent treatment cycles will not be rated as dose-limiting toxicity. If methotrexate and cytarabine cannot be escalated beyond the first dose level, the lenalidomide dose will be reduced to 20 mg with possible further reduction to 15 mg (cf. sections 9.2 and 9.3).

Depending on previous treatment history patients will vary in their tolerance to the LeMLAR regimen. To provide maximum therapeutic benefit, the doses of methotrexate and cytarabine will be escalated within individual patients after cycles 2 and 4 provided no dose limiting toxicities occurred in previous cycles, i.e. patients treated on dose level 1 in cycles 1 and 2 may reach dose level 2 in cycles 3 and 4 and dose level 3 in cycles 5 and 6; patients treated on dose level 2 in cycles 1 and 2 may reach dose level 3 in cycles 3 and 4 and dose level 4 in cycles 5 and 6, etc. Doses in cycles 3 to 6 will be escalated using the levels defined for phase I dose escalation. Dose escalation in individual patients in cycles 3 to 6 will not be used to determine dose-limiting toxicity. This will only be done in cycles 1 and 2.

Phase II
A total of 20 patients will be treated at the maximum tolerated dose level. If in phase I three patients were treated at the maximum tolerated dose, another 17 will be added in phase II. If six patients were treated, another 14 will be included in phase II.

4.2. Study Design Rationale

The phase I part of the study follows a classical 3 + 3 design. Dose-limiting toxicity will only be determined in cycles 1 and 2, because treatment tolerance is likely to decrease from cycle to cycle and toxicities encountered in late cycles should not prompt unnecessary dose reductions in early cycles.

To ensure maximum therapeutic benefit even at low dose levels, the doses of methotrexate and cytarabine may be escalated in individual patients after every other treatment cycle provided no dose-limiting toxicity occurred in previous cycles.

The main goal of the phase II part of the study is to assess the therapeutic efficacy of the LeMLAR regimen at its maximum tolerated dose. In addition the safety data base of the protocol will be expanded.

5. STUDY DURATION

Expected accrual time (first patient in to last patient in):	36 months
Duration of treatment (from last patient in to last patient last visit):	6 months
Duration of follow-up (from last patient last visit to end of trial):	12 months

The results of the phase I part of the trial will be summarized and submitted as an interim report to Celgene. Initiation of phase II will require reliable data defining the maximum tolerated dose and demonstration of clinical activity in at least some of the patients treated in phase I.

Although the duration of follow-up within the funding period of the trial is confined to 12 months, all patients will be followed up until death in the setting of regular patient care. This will guarantee to assess survival times, late toxicities and secondary malignancies.

6. STUDY POPULATION

6.1. Number of Subjects and Sites

Number of subjects in phase I:	3 patients per dose level, if no dose-limiting toxicity occurs<br>6 patients per dose level, if one dose-limiting toxicity occurs
Number of subjects in phase II:	20 patients treated at the maximum tolerated dose (including 3 or 6 patients from phase I)
Number of sites:	3 – 6 tertiary treatment centers in Germany

6.2. Inclusion Criteria

Subjects must satisfy the following criteria to be enrolled in the study:

- Age ≥ 18 years
- Performance status ECOG 0 - 3
- First or subsequent relapse or refractoriness of a biopsy-proven CD20-positive aggressive B cell lymphoma (excluding mantle cell lymphoma)
- Measurable disease
- Ineligibility or unwillingness to undergo high-dose chemotherapy with autologous stem cell transplantation
- Ability to understand the aim of the study and act accordingly
- Subjects should be instructed never to give lenamidomide to another person and to return any unused capsules to the study doctor at the end of treatment.
- Females of childbearing potential enrolled in this protocol must agree to use two reliable forms of contraception simultaneously or to practice complete abstinence from heterosexual contact during the following time periods related to this study: 1) for at least 28 days before starting study drug; 2) while participating in the study; 3) during dose interruptions; and 4) for at least 28 days after study treatment discontinuation. For detailed instructions on the prevention of pregnancies during lenalidomide treatment please refer to Appendix 6 which contains the actual version of the Lenalidomide Pregnancy Prevention Risk Management Plan including the sections “Lenalidomide Risks of Fetal Exposure, Pregnancy Testing Guidelines and Acceptable Birth Control Methods”, “Lenalidomide Education and Counseling Guidance Document” (to be

completed by the investigator prior to each dispensing of lenalidomide study treatment), and "Lenalidomide Information Sheet" (in German; to be handed to the patient).

Signed informed consent (cf. Appendix 5)

6.3. Exclusion Criteria

The presence of any of the following will exclude a subject from enrolment:

Central nervous system relapse of aggressive lymphoma

Any significant medical condition, laboratory abnormality, or psychiatric illness that would prevent the subject from participating in the study

Any condition including the presence of laboratory abnormalities which places the subject at unacceptable risk if he/she were to participate in the study

Any condition that confounds the ability to interpret data from the study

Inadequate organ function not related to aggressive lymphoma:
- neutrophils < 1.0/nl
- platelets < 75/nl
- creatinine clearance < 60 ml/min
- bilirubin ≥ 2,5 mg/dl
- serum AST/GOT or ALT/GPT ≥ 4 x upper limit of normal

Active viral hepatitis (HBV, HCV), HIV infection, any other uncontrolled infection

Pregnancy and nursing period

7. IDENTIFICATION AND REGISTRATION OF PATIENTS

Appropriate patients for inclusion will be identified at each site. After verifying the inclusion and exclusion criteria and obtaining written informed consent the patient will be registered at the LeMLAR trial office:

Priv.-Doz. Dr. Andreas Hüttmann
PETAL Trail Office / Department of Hematology
University Hospital Essen
University of Duisburg-Essen
Hufelandstraße 55
45122 Essen
Germany
Telefone: 0201-723-1861
Telefax: 0201-723-5189
E-mail: petal@uk-essen.de

by providing the following information on the registration form:

- trial site and number*
- sequential patient number at that site*
- confirmation that patient conforms to eligibility criteria
- confirmation that no exclusion criteria apply
- name and address of pathologist

* The trial site will give each patient a 5-digit code number consisting of the 3-digit site number and – separated by a hyphen – the 2-digit sequential patient number at that site (e.g. the fifth patient recruited at the University Hospital Essen would be given the code number 401-05). The patient's code number will be recorded at the trial site in the subject identification code list (cf. Appendix 8).

After obtaining the registration form the trial office will notify the trial site of the dose level to which the patient is assigned.

8. PROCEDURES

In patients with suspected relapse, tumor biopsies are mandatory to demonstrate active CD20-positive lymphoma. In patients with refractory disease, biopsies should also be performed whenever possible without major surgery. The biopsies will be evaluated by a local pathologist, then sent to a reference pathologist who will confirm the diagnosis and perform an in-depth analysis of the lymphoma. In diffuse large B cell lymphomas, part of the biopsy specimens will be used to determine the cell of origin (germinal center B cell [GCB] versus non-germinal center B cell / activated B cell [ABC]). These analyses will be used to correlate clinical observations with biological features of the lymphomas.

The study-related procedures are summarized in the table of events in Appendix 1.

8.1. Pre-Treatment Staging

<u>Mandatory investigations</u>

- patient history
  (date of first diagnosis, previous treatment history with dates, types and results of chemo- and/or radiotherapy episodes; current onset of symptoms, B symptoms; cf. Appendix 2)
- physical examination, vital signs, height, weight, performance status
  (ECOG; cf. Appendix 3)
- laboratory tests
  (hematogram with differential blood cell count, sodium, potassium, calcium, creatinine with calculation of creatinine clearance, uric acid, bilirubin, alkaline phosphatase, gamma-glutamyltransferase, aminotransferases (AST/GOT, ALT/GPT), lactate dehydrogenase, total serum protein, serum protein electrophoresis, albumin, glucose, C-reactive protein, antibodies to human immunodeficiency virus (HIV), hepatitis B virus (HBV) and hepatitis C virus (HCV), pregnancy test with a minimum sensitivity of 25 mIU/ml in woman of childbearing potential)
- imaging studies
  - computed tomography (CT) of neck, thorax, abdomen and pelvis
  - thoracic X-ray in two planes
  - ultrasonography of the abdomen
- biopsies
  - tumor biopsy (mandatory in relapse, recommended in refractory disease)
  - bone marrow biopsy
- cardiopulmonary investigations
  - electrocardiogram (ECG)

- echocardiography (with determination of ejection fraction)

Depending on the clinical situation additional investigations may be necessary (e.g. magnetic resonance imaging, positron emission tomography, endoscopy, lumbar puncture, laboratory investigations).

Based on the pre-treatment staging procedures disease stage and risk group will be defined according to the Ann Arbor classification (cf. Appendix 2) and International Prognostic Index (IPI; cf. Appendix 4), respectively (16,17).

8.2. Investigations in the Course of Treatment

At each scheduled visit for the administration of methotrexate, cytarabine or rituximab, i.e. on days 1, 8, and 15 of each cycle, and additionally on day 22 of each cycle, the following investigations are mandatory:

- patient history (fever, infections, other adverse events, B symptoms)
- vital signs, weight
- laboratory tests
  (hematogram with differential blood cell count, sodium, potassium, calcium, creatinine with calculation of creatinine clearance, uric acid, bilirubin, alkaline phosphatase, gamma-glutamyltransferase, aminotransferases (AST/GOT, ALT/GPT), lactate dehydrogenase, C-reactive protein)

Depending on the clinical situation (e.g. cytopenias, infections) additional investigations or visits may be necessary (e.g. physical examination, imaging studies, laboratory investigations; additional visits in case of severe cytopenias).

In women of childbearing potential a pregnancy test with a minimum sensitivity of 25 mIU/ml must be performed at predefined intervals (10-14 days and 24 hours before the first dose of lenalidomide, weekly within the first 4 weeks of treatment, 4-weekly (2-weekly in case of irregular menstrual cycle) in the subsequent study period, immediately after and 4 weeks after the last treatment cycle).

8.3. Interim Staging (after the first two treatment cycles)

- patient history (B symptoms, adverse events)
- physical examination, vital signs, weight, performance status (ECOG)
- laboratory tests (as for pre-treatment staging except HIV, HBV and HCV serology)
- imaging studies
  - computed tomography of neck, thorax, abdomen and pelvis
- biopsies
  - bone marrow biopsy (only in case of infiltration at pre-treatment staging)

8.4. End-of-Treatment Staging (4 weeks after the end of the last treatment cycle)

- patient history (B symptoms, adverse events)
- physical examination, vital signs, weight, performance status (ECOG)
- Laboratory tests (as for pre-treatment staging except HIV, HBV and HCV serology)
- imaging studies

- computed tomography of neck, thorax, abdomen and pelvis
- thoracic X-ray in two planes
- ultrasonography of the abdomen

- biopsies
    - bone marrow biopsy (only in case of infiltration at pre-treatment and interim staging)

8.5. Follow-up

Follow-up visits will be performed quarterly during the first two years after the end of therapy, 6-monthly during the subsequent 3 years and yearly thereafter. Mandatory investigations include:

- patient history (B symptoms, adverse events)
- physical examination, vital signs, weight, performance status (ECOG)
- laboratory tests (as for pre-treatment staging except HIV, HBV and HCV serology)
- imaging studies
    - thoracic X-ray in two planes
    - ultrasonography of the abdomen

Additional investigations (e.g. imaging studies, biopsies) may be required in case of suspected relapse, secondary malignancy or late toxicities.

9. DESCRIPTION OF STUDY TREATMENTS

9.1. Description of Investigational Products

The following section contains a brief description of the mechanisms of action, modes of application, side effects, mandatory comedications and special safety issues of the drugs used in the LeMLAR trial. **For a more comprehensive description refer to the product information sheets in Appendix 9.**

9.1.1. Cytarabine (e.g. ARA-cell®)

Mechanism of action
Antimetabolite (pyrimidine antagonist), inhibition of DNA synthesis by competitive inhibition of DNA polymerase, incorporation into DNA, induction of DNA strand breaks

Mode of application
Intravenous bolus injection over a 5 to 10 minute period

Major side effects
Myelosuppression, nausea, vomiting, alopecia, skin reactions, hepatotoxicity

Mandatory comedications
None

Special safety issues
Cytarabine must not be mixed with methotrexate in the same syringe (*in vitro* incompatibility).

9.1.2. Lenalidomide (Revlimid®)

Mechanism of action
Immune modulation, antiangiogenesis, modulation of the microenvironment, direct anti-tumor activity, inhibition of tumor necrosis factor alpha, vascular endothelial growth factor and nuclear factor kappa B

Mode of application
Oral

Formulation, storage and preparation
The study drug lenalidomide is provided by the pharmaceutical company Celgene and can be ordered for the six cycles of the LeMLAR protocol. The agent is provided as 25-mg capsules for oral administration within this trial (10-mg or 15-mg capsules are provided if, at a lenalidomide dose of 25 mg, methotrexate and cytarabine cannot be escalated beyond the first dose level; cf. sections 9.2 and 9.3). Each capsule contains in addition to lenalidomide the following inactive ingredients: anhydrous lactose, microcrystalline cellulose, croscarmellose sodium, and magnesium stearate. Study drug will be packaged in blister containing study capsules for 21 days.

Side effects
Lenalidomide is associated with anemia, neutropenia, febrile neutropenia, thrombocytopenia and pancytopenia. Grade 3/4 neutropenia and thrombocytopenia are the most common, dose-limiting adverse events associated with the administration of lenalidomide.

Lenalidomide, in combination with dexamethasone, has been associated with an increased incidence in thrombotic or thromboembolic events, including deep vein thrombosis, pulmonary embolism, thrombosis, and thromboembolism, particularly in patients with multiple myeloma receiving concomitant therapy with an erythropoietic agent.

Constipation, diarrhea, nausea, and vomiting were the most commonly reported gastrointestinal adverse events during treatment with lenalidomide. Common events like atrial fibrillation, myocardial infarction, and congestive heart failure have been reported with the use of lenalidomide from clinical studies. Treatment-emergent adverse events of infections specifically pneumonia are commonly seen with lenalidomide. Vigilance for the signs and symptoms of infections should be practiced in patients treated with lenalidomide. An increase in secondary malignancies was observed in patients with multiple myeloma undergoing high-dose chemotherapy with subsequent lenalidomide maintenance therapy.

Tumour lysis syndrome and tumor flare syndrome have been observed in patients with chronic lymphocytic leukemia who were treated with lenalidomide. Patients at risk for tumor lysis syndrome are those with high tumor burden prior to treatment. Rarely rhabdomyolysis has been observed with lenalidomide. Angioedema and serious dermatologic reactions including Stevens-Johnson syndrome and toxic epidermal necrolysis have been reported rarely with lenalidomide during commercial use. These events have the potential to be fatal.

Mandatory comedications
Prophylaxis against thromboembolism: enoxaparin 40 mg s.c. daily (to be stopped if platelets drop below 50/nl)
Reliable contraceptive measures for women of childbearing potential and men with female partners of childbearing potential

Special safety issues (**pregnancy warning; for more detailed informations cf. Appendix 6**)
Lenalidomide is structurally related to thalidomide. Thalidomide is a known teratogenic substance that causes severe life-threatening birth defects. Findings from an ongoing embryofetal development study in animals indicate that lenalidomide produced malformations in

the offspring of female monkeys who received the drug during pregnancy. If lenalidomide is taken during pregnancy, a teratogenic effect in humans cannot be ruled out.

As a result lenalidomide is subject to controlled distribution.

9.1.3. Methotrexate (e.g. Methotrexat medac) and Folinic Acid (Leucovorin®)

<u>Mechanism of action</u>
Antimetabolite (folic acid antagonist), inhibition of dihydrofolate reductase, inhibition of de novo purine synthesis

<u>Mode of application</u>
Intravenous bolus injection over a 5 to 10 minute period

<u>Major side effects</u>
Orointestinal mucositis, hepatotoxicity, pneumonitis, pulmonary fibrosis, immunosuppression

<u>Dosage</u>
Because the major route of methotrexate elimination is renal, its dose must be adapted to renal function. To this end the creatinine clearance will be calculated using the Cockcroft-Gault formula (18) which is available at the Internet under http://www.nephron.com/cgi-bin/CGSI.cgi. The following guidelines for dose adjustments are given in the methotrexate product information sheet (cf. Appendix 9):

Creatinine clearance > 80 ml/min:	full dose according to protocol
Creatinine clearance = 80 ml/min:	75% of dose indicated in the protocol
Creatinine clearance = 60 ml/min:	63% of dose indicated in the protocol
Creatinine clearance < 60 ml/min:	no methotrexate

Patients with a baseline creatinine clearance < 60 ml/min cannot participate in the LeMLAR trial (exclusion criterion; cf. section 6.3.). If the creatinine clearance drops below 60 ml/min during the first two treatment cycles and does not recover to a value ≥ 60 ml/min within 3 days (starting on day 8 of each cycle), 6 days (starting on day 15) or 7 days (starting on day 1 of the subsequent cycle), respectively, dose-limiting toxicity is reached (cf. section 9.3.)

<u>Mandatory comedications</u>
Folinic acid (leucovorin) rescue: 45 mg leucovorin p.o., 4 doses given every 6 hours, starting 24 hours after methotrexate injection

<u>Special safety issues</u>
Methotrexate retention in third space: prolonged action in patients with pleural effusions or ascites (must be voided before methotrexate use)
Interactions with numerous drugs may lead to increased methotrexate toxicity (e.g. acetylsalicylic acid - increased bio-availability; proton pump inhibitors - delayed renal elimination; non-steroidal antirheumatic drugs - elevated serum levels). For a complete list of interactions refer to “Rote Liste 2012; Rote Liste® Service GmbH, Frankfurt/Main). Whenever possible comedication with drugs interacting with methotrexate should be avoided.
Methotrexate must not be mixed with cytarabine in the same syringe (*in vitro* incompatibility).

9.1.4. Rituximab (MabThera®)

<u>Mechanism of action</u>
Monoclonal antibody to CD20, cytotoxicity by binding to CD20 and activation of complement (complement-dependent cytotoxicity) and/or phagocytes (antibody-dependent cellular

cytotoxicity)

Mode of application
Intravenous infusion

First dose:	premedication	1000 ml Ringer's solution<br>1 g paracetamol p.o.<br>2 mg clemastine (Tavegil®) i.v.<br>or 4 mg dimetinden(Fenistil®) i.v.<br>100 mg prednisone i.v.
	infusion rate	first hour: 50 mg<br>if well tolerated, stepwise (every 30 minutes) increase of infusion rate to 100 – 200 – 300 – 400 mg per hour

Subsequent doses (only if first dose was well tolerated; otherwise repeat first dose schedule)

	premedication	1000 ml Ringer's solution<br>1 g paracetamol p.o.<br>2 mg clemastine (Tavegil®) i.v.<br>or 4 mg dimetinden(Fenistil®) i.v.
	total infusion time	90 minutes

Major side effects
Fever, rigor, hypotension, bronchospasm (especially during first infusion), late neutropenia

Mandatory comedications
See above (premedication)

Special safety issues
Monitoring of vital signs during infusion

9.2. Treatment Administration and Schedule

Treatment plan (the LeMLAR protocol):

Lenalidomide	25 mg p.o.*	days 1 – 21 (28 day cycles)
Methotrexate	30 – 60 – 90 – 120 – 150 mg/m² i.v. bolus	days 1, 8, 15
Leucovorin	4 x 45 mg p.o. (every 6 hours)	days 2, 9, 16
Cytarabine (Ara-C)	75 – 150 – 225 – 300 – 375 mg/m² i.v. bolus	days 1, 8, 15
Rituximab	375 mg/m² i.v. infusion	day 1

* stepwise reduction to 20 mg or 15 mg if methotrexate and cytarabine cannot be escalated beyond the first dose level

Lenalidomide will be given at a fixed dose of 25 mg per day for the first three weeks of each four-week treatment cycle. Methotrexate and cytarabine will be given as intravenous bolus injections over a 5 to 10 minute period on days 1, 8 and 15. The first dose of leucovorin will be taken orally 24 hours after methotrexate injection. For reduction of methotrexate dose in case of impaired renal function cf. section 9.1.3. Rituximab will be infused on day 1 according to the schedule outlined in section 9.1.4. Treatment cycles will be repeated every 28 days for a maximum of six cycles.

The dose level below the level where dose-limiting toxicity is observed defines the maximum tolerated doses of methotrexate and cytarabine. However, if, at a lenalidomide dose of 25 mg,

methotrexate and cytarabine cannot be escalated beyond the first dose level, the lenalidomide dose will be reduced to 20 mg, and the methotrexate/cytarabine dose level producing dose-limiting toxicity will be repeated at a lenalidomide dose of 20 mg. If, at a lenalidomide dose of 20 mg, methotrexate and cytarabine cannot be escalated beyond the first dose level, the lenalidomide dose will be reduced to 15 mg, and the methotrexate/cytarabine dose level producing dose-limiting toxicity will be repeated with a lenalidomide dose of 15 mg. If, at a lenalidomide dose of 15 mg, methotrexate and cytarabine cannot be escalated beyond the first dose level, dose limiting toxicity is reached and the maximum tolerated dose is the highest dose of lenalidomide able to be combined with the first dose level of methotrexate and cytarabine in a 3 + 3 phase I design.

To prevent thromboembolic complications enoxaparin will be given s.c. at a dose of 40 mg daily throughout the treatment period. Enoxaparin will be discontinued if the platelet count drops below 50/nl.

To prevent delays due to neutropenia G-CSF will be given if neutrophil levels drop below 0.5/nl, and continued until the neutrophil count rises above 1.0/nl. Suitable G-CSF products and dosages include filgrastim 5 µg/kg body-weight and lenograstim 150 µg/m² body-surface area s.c. once daily.

Before the first cycle the patient will be assigned by the trial office to one of five dose levels of methotrexate and cytarabine (see below). If no dose-limiting toxicity occurs, doses of methotrexate and cytarabine will be increased after every other cycle according to the scheme detailed below. In case of dose-limiting toxicity the dose of the compound(s) most likely responsible will be reduced.

9.3. Treatment Assignment in Phase I and Phase II

Phase I

The goal of the phase I part of the study is to determine the maximum tolerated doses of methotrexate and cytarabine able to be combined with once-per-cycle rituximab and full-dose 3-weeks-on/1-week-off lenalidomide in 28-day treatment cycles. Methotrexate and cytarabine will be given three times per cycle, preferably on days 1, 8 and 15. If short-term toxicity precludes once-per-week dosing, treatment may be delayed by a maximum of 3 days, e.g. the second injection of methotrexate and cytarabine may be delayed until day 11 and the third injection until day 21. The doses of methotrexate and cytarabine will be adjusted to permit administration of three doses per cycle without the need to postpone the subsequent treatment cycle by more than 7 days.

Dose limiting toxicity will be determined by increasing the doses of methotrexate and cytarabine in sequential patient cohorts in a 3 + 3 design. Because tolerance to lenalidomide and cytotoxic agents are likely to decrease with increasing numbers of treatment cycles, evaluation of dose-limiting toxicity will be confined to the first two cycles. The requirement for dose reductions in subsequent treatment cycles will not be rated as dose-limiting toxicity.

If no dose-limiting toxicity is observed in 3 patients treated at the same dose level, the next 3 patients will be treated at the next higher dose level. If a single patient experiences dose-limiting toxicity, another 3 patients will be treated at the same dose level. If no more than one dose-limiting toxicity is observed in 6 patients treated at that level, the next 3 patients will be treated at the subsequent dose level. If two or more of 3 – 6 patients treated at the same level experience dose-limiting toxicity, dose escalation will be stopped. The dose level below the level where dose-limiting toxicity was observed defines the maximum tolerated dose.

The dose level below the level where dose-limiting toxicity is observed defines the maximum tolerated doses of methotrexate and cytarabine. However, if, at a lenalidomide dose of 25 mg,

methotrexate and cytarabine cannot be escalated beyond the first dose level, the lenalidomide dose will be reduced to 20 mg, and the methotrexate/cytarabine dose level producing dose-limiting toxicity will be repeated at a lenalidomide dose of 20 mg. If, at a lenalidomide dose of 20 mg, methotrexate and cytarabine cannot be escalated beyond the first dose level, the lenalidomide dose will be reduced to 15 mg, and the methotrexate/cytarabine dose level producing dose-limiting toxicity will be repeated with a lenalidomide dose of 15 mg. If, at a lenalidomide dose of 15 mg, methotrexate and cytarabine cannot be escalated beyond the first dose level, dose limiting toxicity is reached and the maximum tolerated dose is the highest dose of lenalidomide able to be combined with the first dose level of methotrexate and cytarabine in a 3 + 3 phase I design.

The toxicity profiles of methotrexate (mucositis, hepatotoxicity) and cytarabine (hematotoxicity, hepatotoxicity) differ. In order to determine the maximum tolerated dose of each compound, further dose escalation of one or other drug may be required after the maximum tolerated dose of the methotrexate/cytarabine combination (as defined in dose levels 1 – 5, see below) has been determined. If dose-limiting hematotoxicity occurs without concomitant dose-limiting mucositis or hepatotoxicity, the dose of methotrexate may be escalated without concomitant increase of the cytarabine dose. Conversely, if severe mucositis occurs without concomitant dose-limiting hematotoxicity or hepatotoxicity, the dose of cytarabine may be escalated without concomitant increase of the methotrexate dose. Dose modifications of individual drugs will be made by the coordinating principal investigator according to the observed toxicities using the levels defined for the methotrexate/cytarabine combination.

Toxicities observed during the first two treatment cycles will be reported to the trial office within 7 days after their occurrence (in case of serious adverse events within 24 hours; cf. section 11.1.). Based on the predefined criteria listed below the coordinating principal investigator and the trial coordinator will decide whether or not a toxicity fulfils the requirements of a dose-limiting toxicity. After inclusion of up to 6 patients on the same dose level the coordinating principal investigator and the trial coordinator will decide according to the principles outlined in this protocol whether it is safe to move on to the next dose level. If rating a toxicity as dose-limiting is equivocal and/or no agreement is obtained between the coordinating principal investigator and the trial coordinator, the principal investigators of all participating trial sites will be included in the decision making process. Stepping-up to the next dose level will require more votes to be in favor of than against dose escalation.

Dose-limiting toxicities are:

- any of the following on the day of methotrexate/cytarabine injection (day 8 + ≤ 3 days / day 15 + ≤ 6 days of the first or second treatment cycle; day 1 of the second or third cycle which is equivalent to day 29 + ≤ 7 days of the previous cycle):
    - neutrophils < 500/µl
    - platelets < 25.000/µl
    - creatinine clearance < 60 ml/min
    - bilirubin ≥ 3,0 mg/dl
    - serum AST/GOT or ALT/GPT ≥ 6 x upper limit of normal
    - mucositis grade 3 or 4
- requirement for dose reduction of methotrexate/cytarabine in the first or second treatment cycle (cf. section 9.4)
- fewer than 21 days of lenalidomide in the first or second treatment cycle

- toxicity-related delay of second or third treatment cycle by more than 7 days
- any other toxicity preventing continuation of therapy according to protocol in the first or second treatment cycle (except allergic reactions)

Depending on previous treatment history patients will vary in their tolerance to the LeMLAR regimen. To provide maximum therapeutic benefit, the doses of methotrexate and cytarabine will be escalated within individual patients after cycles 2 and 4 as detailed below provided no dose limiting toxicities occurred in previous treatment cycles. Dose escalation in cycles 3 to 6 in individual patients will not be used to determine dose-limiting toxicity. This will only be done in cycles 1 and 2.

Patients should receive 6 treatment cycles unless tumor progression, unacceptable toxicity or treatment intolerance occurs. Treatment intolerance includes physician or patient preference to discontinue or change treatment in a manner not compatible with the protocol. If treatment according to the LeMLAR protocol is prematurely stopped, its result must be documented by the procedures outlined in section 8.4 (end-of-treatment staging).

Definition of dose levels of methotrexate and cytarabine (cohorts of 3 – 6 patients):

Level 1
Cycles 1 – 2: methotrexate 30 mg/m², cytarabine 75 mg/m²
Cycles 3 – 4: if no dose-limiting toxicity occurs in cycles 1 and 2:
methotrexate 60 mg/m², cytarabine 150 mg/m²
Cycles 5 – 6: if no dose-limiting toxicity occurs in cycles 3 and 4:
methotrexate 90 mg/m², cytarabine 225 mg/m²

Level 2
Cycles 1 – 2: methotrexate 60 mg/m², cytarabine 150 mg/m²
Cycles 3 – 4: if no dose-limiting toxicity occurs in cycles 1 and 2:
methotrexate 90 mg/m², cytarabine 225 mg/m²
Cycles 5 – 6: if no dose-limiting toxicity occurs in cycles 3 and 4:
methotrexate 120 mg/m², cytarabine 300 mg/m²

Level 3
Cycles 1 – 2: methotrexate 90 mg/m², cytarabine 225 mg/m²
Cycles 3 – 4: if no dose-limiting toxicity occurs in cycles 1 and 2:
methotrexate 120 mg/m², cytarabine 300 mg/m²
Cycles 5 – 6: if no dose-limiting toxicity occurs in cycles 3 and 4:
methotrexate 150 mg/m², cytarabine 375 mg/m²

Level 4
Cycles 1 – 2: methotrexate 120 mg/m², cytarabine 300 mg/m²
Cycles 3 – 6: if no dose-limiting toxicity occurs in cycles 1 and 2:
methotrexate 150 mg/m², cytarabine 375 mg/m²

Level 5
Cycles 1 – 6: methotrexate 150 mg/m², cytarabine 375 mg/m²

Further dose escalation is not planned because the doses of level 5 are 25 % above those of the original COMLA protocol in which methotrexate and cytarabine were given in conjunction with cyclophosphamide and vincristine (13,14).

Phase II

The results of the phase I part of the trial will be summarized and submitted as an interim report to Celgene. Initiation of phase II will require reliable data defining the maximum tolerated dose and demonstration of clinical activity in at least some of the patients treated in phase I.

A total of 20 evaluable patients will be treated at the maximum tolerated dose level. If in phase I three patients were treated at the maximum tolerated dose, another 17 will be added in phase II. If six patients were treated in phase I, another 14 will be included in phase II.

Patients in the phase II part will receive a maximum of 6 treatment cycles. If no dose-limiting toxicity occurs in the first two cycles, the doses of methotrexate and cytarabine may be escalated in cycles 3 and 4, and, in case of no toxicities in cycles 3 and 4, again in cycles 5 and 6. Treatment will be stopped prematurely in case of tumor progression, unacceptable toxicity, intolerance or physician or patient preference. In case of premature discontinuation treatment result should be documented by the procedures outlined in section 8.4 (end-of-treatment staging).

9.4. Temporary Discontinuation of Treatment and Dose Reduction

Treatment will be halted in the event of neutrophils < 300/µl without fever, neutrophils < 1.000/µl with fever, platelets < 15.000/µl, infections, severe cutaneous or allergic reactions, venous thromboembolism or any other event that in the opinion of the investigator precludes continuation of treatment. Upon resolution of hematologic toxicity (to values above those defined above) and any clinical signs of infection treatment may be resumed according to clinical judgement. In case of severe cutaneous or allergic reactions or thromboembolism treatment will be discontinued permanently.

To prevent delays due to neutropenia G-CSF will be started when neutrophil levels drop below 0.5/nl, and continued until the neutrophil count rises above 1.0/nl. Suitable G-CSF products and dosages include filgrastim 5 µg/kg body-weight and lenograstim 150 µg/m² body-surface area s.c. once daily. Red blood cells and platelets will be substituted according to established guidelines.

In the event of severe hematotoxicity blood counts should be monitored at least twice a week. After resolution of hematotoxicity and/or infection, treatment should be resumed at the same lenalidomide dose as used before the event and continued for a total of 21 days per cycle. The doses of methotrexate and cytarabine will be reduced by one dose level if neutrophils or platelets fail to recover to levels of 500/µl or 25.000/µl, respectively, on the planned day of injection. In treatment cycles 1 or 2 this will be recorded as dose-limiting toxicity. Dose reductions in cycles 3 – 6 will not be counted as dose-limiting toxicity.

9.5. Packaging, Labeling, Accountability and Disposal of Lenalidomide

Lenalidomide will be provided to the investigators free of charge by Celgene and will be labeled according to the legal requirements. The sponsor is responsible for the drug reconciliation at every participating site. The investigator or subinvestigator at each site will return all unused or expired lenalidomide capsules for disposal to his local pharmacy. The local pharmacy will provide a written protocol (IMPD Log) about the used, unused and returned drug prior to destruction and a written confirmation that the capsules were destroyed. A copy of the IMPD Log and destruction protocol will be sent to Celgene.

10. STATISTICAL ANALYSES

10.1. Study Endpoints Definition

Dose-limiting toxicity	cf. section 9.3
Maximum tolerated dose	dose level below the level at which more than one patient in a cohort of 3 – 6 patients was affected by dose-limiting toxicity

Treatment response will be assessed using the International Workshop Criteria for Non-Hodgkin's Lymphomas (19):

Overall response rate	percentage of complete and partial remissions combined
Complete remission	complete disappearance of all detectable clinical and radiographic evidence of disease and disappearance of all disease-related symptoms if present before therapy, and normalization of those biochemical abnormalities (e.g., lactate dehydrogenase) definitely assignable to lymphoma
Complete remission (unconfirmed)	complete remission except for a residual lymph node mass greater than 1.5 cm in greatest transverse diameter that has regressed by more than 75% in the sum of the product of the diameters
Partial remission	≥ 50% decrease in the sum of the products of the diameters of the six largest dominant nodes or nodal masses
Stable disease	less than a partial remission but not progressive disease
Progressive disease	≥ 50% increase from nadir in the sum of the product of the diameters of any previously identified abnormal node or appearance of any new lesion during or at the end of therapy
Relapse	appearance of any new lesion or increase by ≥ 50% in the size of previously involved sites in patients in complete remission or complete remission (unconfirmed)
Relapse rate	percentage of patients relapsing after achieving a complete remission or complete remission (unconfirmed)
Progression-free survival	time from entry onto trial to disease progression or death from lymphoma
Event-free survival	time from entry onto trial to treatment failure or death from any cause; treatment failure is defined as failure to achieve complete or partial remission, premature treatment termination due to toxicity or intolerance without achievement of complete or partial remission, or disease progression
Disease-free survival	time from achievement of complete remission or complete remission (unconfirmed) to relapse
Overall survival	time from entry onto trial to death from any cause
Secondary malignancy	any malignancy manifesting after inclusion in the trial

10.2. Study Population Definitions

The intent-to-treat population (adapted to this one-arm non-randomized trial setting) will include all registered patients with informed consent that fulfil all inclusion and no exclusion criteria. The

safety analysis set will include all patients with informed consent that received at least one dose of treatment and for whom at least one post-baseline observation is available. The per protocol population will include all patients with informed consent that received at least two treatment cycles and underwent end-of-treatment staging.

10.3. Sample Size and Power Considerations

The phase I part of the study follows a 3 + 3 design. If no toxicity occurs in a cohort of 3 patients or no more than one patient experiences toxicity in a cohort of 6 patients, the next patient cohort will be treated with the next higher dose level. This is a compromise to keep the probabilities of premature discontinuation of dose escalation or unjustified acceptance of a toxic dose level low. The number of patients required will depend on the tolerability of the LeMLAR regimen. The maximum number of patients to be treated with the pre-specified dose escalation scheme (cf. section 9.3) will be 30 (5 dose levels, maximally 6 patients per dose level). If methotrexate and cytarabine cannot be escalated beyond the first dose level, the dose of lenalidomide will be reduced to 20 mg with possible further reduction to 15 mg. This will require a maximum of 12 additional patients. Therefore, the maximum number of patients to be included in the phase I part of the trial is 42.

The estimation of sample size in the phase II part is performed by a single-stage procedure as described in (20,21). Previous experience has shown that the overall response rate (complete and partial remissions combined) is 20% with lenalidomide alone (6,9). Defining p_0 as the proportion of responses below which treatment does not warrant further investigation and p_a as the proportion of responses beyond which the regimen should be explored further, we set $p_0 = 0.2$ and $p_a = 0.4$. We will test the null hypothesis H_0: $p \leq p_0$ against the alternative hypothesis H_1: $p \geq p_a$ where p denotes the true overall response rate. In addition we set the significance level α (two-sided) at 0.05 and the power at $1-\beta = 0.40$. For these specifications the number of patients necessary for the analysis is 18 and the number of overall responses needed is 8 to reject H_0 in favor of H_1. With an expected drop-out rate of 10% the required sample size is 20.

10.4. Background and Demographic Characteristics

Subjects' age, height, weight, and baseline characteristics will be summarized using descriptive statistics, while gender and other categorical variables will be provided using frequency tabulations. Medical history data will be summarized using frequency tabulations by system organ class and preferred term.

10.5. Subject Disposition

Subject disposition (analysis population allocation, entered, discontinued, along with primary reason for discontinuation) will be summarized using frequency and percent for both treatment and follow-up phases. A summary of subjects enrolled by site will be provided. Protocol deviations will be summarized using tables.

10.6. Efficacy Analysis

All principal analyses will pertain to the intent-to-treat population (as defined above and adapted to this one-arm non-randomized trial setting). The overall response rate will be estimated for all intent-to-treat patients treated at the maximum tolerated dose level by dividing the number of patients with a response at end-of-therapy staging by the number of all patients. The same analysis will be done for the per protocol population.

The results obtained (remission and relapse rates, survival parameters, toxicity) will be compared with the original COMLA protocol (14), reports of rituximab-containing salvage regimens not containing lenalidomide and any lenalidomide-based treatment strategies for patients with relapsed or refractory CD20-positive aggressive lymphomas not qualifying for high-dose chemotherapy with autologous stem cell transplantation that may be available at the time of the final analysis.

10.7. Safety Analysis

The safety analysis will include a description and tabulation of the frequency and severity of adverse and serious adverse events, suspected unexpected serious adverse reactions, incidence and severity of non-hematological and hematological toxicities grades 3 and 4, rate of treatment-related deaths, secondary malignancies as well as deviations of selected laboratory parameters. Toxicities and adverse events will be graded according to the most recent version of the Common Terminology Criteria for Adverse Events scale of the National Cancer Institute (http://evs.nci.nih.gov/ftp1/CTCAE/CTCAE_4.03_2010-06-14_QuickReference_5x7.pdf).

10.8 Interim Analysis

Serious adverse events and deaths occurring during the study will be continuously monitored by the trial coordinator. No formal criterion for early discontinuation will be defined. An interim analysis will be performed after completion of the dose escalation part of the trial. The results will be the basis for initiation of the phase II part of the study.

11. ADVERSE EVENTS

11.1. Monitoring, Recording and Reporting of Adverse Events

An adverse event (AE) is any noxious, unintended, or untoward medical occurrence that may appear or worsen in a subject during the course of a study. It may be a new intercurrent illness, a worsening concomitant illness, an injury, or any concomitant impairment of the subject’s health, including laboratory test values (as specified by the criteria below), regardless of etiology. Any worsening (i.e., any clinically significant adverse change in the frequency or intensity of a preexisting condition) should be considered an AE. A diagnosis or syndrome should be recorded on the AE report form rather than the individual signs or symptoms of the diagnosis or syndrome. Progression of aggressive lymphoma is not regarded as an AE.

An overdose, accidental or intentional, whether or not it is associated with an AE, or abuse, withdrawal, sensitivity or toxicity to an investigational product should be reported as an AE. If an overdose is associated with an AE, the overdose and adverse event should be reported as separate terms.

All subjects will be monitored for AEs during the study. Assessments may include monitoring of any or all of the following parameters: the subject’s clinical symptoms, laboratory, pathological, radiological or surgical findings, physical examination findings, or other appropriate tests and procedures.

All AEs except secondary malignancies will be recorded by the investigator from the time the subject signs informed consent to end-of-treatment staging, i.e. 4 weeks after the last treatment cycle. Secondary malignancies will be recorded until the subject’s death. AEs and serious adverse events (SAEs) will be recorded on the AE and SAE report forms, respectively, and in the subject’s source documents. All SAEs must be reported to the trial office within 24 hours of the investigator’s knowledge of the event by facsimile, or other appropriate method, using the

SAE report form.

11.2. Evaluation of Adverse Events

The investigator will evaluate all adverse events as to:

11.2.1. Seriousness

A serious adverse event (SAE) is any AE occurring at any dose that:

- results in death;
- is life-threatening (i.e., in the opinion of the investigator, the subject is at immediate risk of death from the AE);
- requires inpatient hospitalization or prolongation of existing hospitalization (hospitalization is defined as an inpatient admission, regardless of length of stay);
- results in persistent or significant disability/incapacity (a substantial disruption of the subject’s ability to conduct normal life functions);
- is a congenital anomaly/birth defect;
- constitutes an important medical event.

Important medical events are defined as those occurrences that may not be immediately life threatening or result in death, hospitalization, or disability, but may jeopardize the subject or require medical or surgical intervention to prevent one of the other outcomes listed above. Medical and scientific judgment should be exercised in deciding whether such an AE should be considered serious.

Second primary malignancies will be monitored as events of interest and must be reported as SAEs. This includes any second primary malignancy, regardless of causal relationship, occurring at any time for the duration of the study, from the time of signing the inform consent until the subject’s death. Events of second primary malignancy are to be reported using the SAE report form and must be considered an important medical event even if no other serious criteria apply; these events must also be documented in the appropriate page(s) of the case report form (CRF) and subject’s source documents. Documentation on the diagnosis of the second primary malignancy must be provided at the time of reporting as a serious adverse event (e.g., any confirmatory histology or cytology results, X-rays, CT scans, etc.).

Events **not considered** to be SAEs are hospitalizations for:

- a standard procedure for protocol therapy administration. However, hospitalization or prolonged hospitalization for a complication of therapy will be reported as an SAE.
- routine treatment or monitoring of the studied indication not associated with any deterioration in condition.
- the administration of blood or platelet transfusion as routine treatment of studied indication. However, hospitalization or prolonged hospitalization for a complication of such transfusion remains a reportable SAE.
- a procedure for protocol/disease-related investigations (e.g., surgery, scans, endoscopy, sampling for laboratory tests, bone marrow sampling). However, hospitalization or prolonged hospitalization for a complication of such procedures remains a reportable SAE.

- hospitalization or prolongation of hospitalization for technical, practical, or social reasons, in absence of an AE.
- a procedure that is planned (i.e., planned prior to starting of treatment on study); must be documented in the source document and the CRF. Hospitalization or prolonged hospitalization for a complication remains a reportable SAE.
- an elective treatment of a pre-existing condition unrelated to the studied indication.
- emergency outpatient treatment or observation that does not result in admission, unless fulfilling other seriousness criteria.

If an AE is considered serious, both the AE and the SAE report form must be completed.

For each SAE, the investigator will provide information on severity, start and stop dates, relationship to investigational product(s), action taken regarding investigational product(s), and outcome.

11.2.2. Severity

For both AEs and SAEs, the investigator must assess the severity of the event. The term “severe” is often used to describe the intensity of a specific event (as in mild, moderate or severe myocardial infarction); the event itself, however, may be of relatively minor medical significance (such as severe headache). This criterion is *not* the same as “serious” which is based on subject/event *outcome* or *action* criteria associated with events that pose a threat to a subject’s life or functioning. Seriousness, not severity, serves as a guide for defining regulatory obligations.

11.2.3. Causality

The investigator must determine the relationship between the administration of investigational product(s) and the occurrence of an AE/SAE as Not Suspected or Suspected as defined below:

Not suspected: The temporal relationship of the adverse event to the investigational product administration makes **a causal relationship unlikely or remote**, or other medications, therapeutic interventions, or underlying conditions provide a sufficient explanation for the observed event.

Suspected: The temporal relationship of the adverse event to the investigational product administration makes **a causal relationship possible**, and other medications, therapeutic interventions, or underlying conditions do not provide a sufficient explanation for the observed event.

11.2.4. Duration

The investigator will provide a record of the start and stop dates of the AE/SAE. The duration of the AE and the SAE may vary within one event. For example, a non-serious AE may begin on 01-Jan. The event will become serious when it meets one of the criteria for seriousness (e.g., the subject is hospitalized on 05-Jan). The SAE will continue until it no longer meets the seriousness criteria (e.g., the subject is discharged on 07-Jan). However, the AE continues until 10-Jan when the event resolves. The AE dates may extend from before and beyond the SAE dates, but not the reverse.

11.2.5. Action Taken

The investigator will report the discontinuation or dose reduction of the investigational product following an AE and report if concomitant and/or additional treatments were given for the AE.

11.2.6. Outcome

All SAEs that have not resolved upon discontinuation of the subject's participation in the study must be followed until recovered, recovered with sequelae, not recovered (death due to another cause) or death (due to the SAE).

11.3. Abnormal Laboratory Values

An abnormal laboratory value is considered to be an AE if the abnormality:

- results in discontinuation from the study;
- requires treatment, modification or interruption of the investigational product dose, or any other therapeutic intervention; or
- is judged to be of significant clinical importance.

Regardless of severity grade, only laboratory abnormalities that fulfil a seriousness criterion need to be documented as a serious adverse event. If a laboratory abnormality is one component of a diagnosis or syndrome, then only the diagnosis or syndrome should be recorded on the AE report form. If the abnormality was not a part of a diagnosis or syndrome, then the laboratory abnormality should be recorded as the AE.

11.4. Pregnancy

11.4.1 Pregnancy Prevention Program

Appendix 6 contains the current version of the Lenalidomide Pregnancy Prevention Risk Management Plan including the sections "Lenalidomide Risks of Fetal Exposure, Pregnancy Testing Guidelines and Acceptable Birth Control Methods", "Lenalidomide Education and Counseling Guidance Document" (to be completed by the investigator prior to each dispensing of lenalidomide study treatment), and "Lenalidomide Information Sheet" (in German; to be handed to the patient).

11.4.2 Reporting procedures for pregnancies

<u>Females of Childbearing Potential</u>
Pregnancies and suspected pregnancies (including a positive pregnancy test regardless of age or disease state) of a female subject occurring while the subject is on study drug, or within one year of the subject's last dose of study drug, are considered events to be reported immediately to the LeMLAR trial office and Celgene. If the subject is on study drug, the study drug is to be discontinued immediately and the subject instructed to return any unused portion of the study drug to the investigator. The pregnancy, suspected pregnancy, or positive pregnancy test must be reported to the LeMLAR trial office, who will inform Celgene immediately by telephone or facsimile using an SAE report form.

The female should be referred to an obstetrician/gynecologist experienced in reproductive toxicity for further evaluation and counseling.

The investigator will follow the female subject until completion of the pregnancy, and must notify the LeMLAR trial office of the outcome of the pregnancy immediately as specified below. The investigator will provide this information as a follow-up to the initial pregnancy report. The LeMLAR trial office will communicate this information immediately to Celgene.

If the outcome of the pregnancy meets the criteria for immediate classification as a SAE (i.e., spontaneous or therapeutic abortion, any congenital anomaly (including that in an aborted fetus), stillbirth, neonatal death), the investigator should follow the procedures for reporting SAEs.

All neonatal deaths that occur within 28 days of birth should be reported, without regard to causality, as SAEs. In addition, any infant death after 28 days that the investigator suspects is related to the *in utero* exposure to the study drug should also be reported.

<u>Male Subjects</u>
Female partners of males taking investigational product should be advised to call their healthcare provider immediately if they get pregnant. The male subject should notify the investigator of his partner's pregnancy and her healthcare provider information. The investigator should ask if the female partner is willing to share information with Celgene Drug Safety and allow the pregnancy related event to be followed up to completion. The investigator will then provide this information to the LeMLAR trial office who will inform Celgene immediately. This concerns any pregnancies or suspected pregnancies (including a positive pregnancy test regardless of age or disease state) occurring in female partners of patients while the patients are still treated with lenalidomide or within 28 days of the patients' last dose of lenalidomide.

11.5. Immediate Reporting of Serious Adverse Events

Any AE that meets any criterion for an SAE requires the completion of an SAE report form in addition to being recorded on the AE report form. All SAEs must be reported to the trial office within 24 hours of the investigator's knowledge of the event by facsimile, or other appropriate method, using the SAE report form. This instruction pertains to initial SAE reports as well as any follow-up reports.

The investigator is required to ensure that the data on these forms is accurate and consistent. This applies to all SAEs including secondary malignancies (regardless of the relationship to the investigational products) that occur during the study (from the time the subject signs informed consent to end-of-treatment staging, i.e. 4 weeks after the last treatment cycle), and those made known to the investigator at anytime thereafter that are suspected of being related to the investigational products. Secondary malignancies will be recorded as SAEs until the subject's death. SAEs occurring after signing informed consent and prior to treatment will be captured.

The SAE report should provide a detailed description of the SAE and include copies of hospital records and other relevant documents. If a subject died and an autopsy was performed, copies of the autopsy report are to be sent to the trial office as soon as these become available. Any follow-up data will be detailed in a subsequent SAE report form and sent to the trial office.

Where required by local legislation, the investigator is responsible for informing the ethics committee of the SAE and providing them with all relevant initial and follow-up information about the event. The investigator must keep copies of all SAE information on file including correspondence with the trial office and the ethics committee.

The trial office will supply Celgene with a copy of all SAEs which involve *exposure* to a Celgene product within 24 hours of being made aware of the event regardless of whether or not the event is listed in the reference document (e.g. investigator's brochure, summary of product

characteristics).The trial office will provide Celgene with a copy of the annual periodic safety report at the time of submission to the Regulatory Authority and Ethics Committee.

11.5.1. Safety Queries

Queries pertaining to SAEs will be communicated from the trial office to the site via facsimile or electronic mail. The response time is expected to be no more than five (5) business days. Urgent queries (e.g., missing causality assessment) may be handled by phone.

11.6. Expedited Reporting of Adverse Events (SUSARs)

For the purpose of regulatory reporting, the coordinating principal investigator will determine the expectedness of events suspected of being related to the investigational products based on the known side effects of the compounds. The trial office will report in an expedited manner to the regulatory authorities and ethics committees concerned, suspected unexpected serious adverse reactions (SUSARs) in accordance with Directive 2001/20/EC and the Detailed Guidance on collection, verification and presentation of adverse reaction reports arising from clinical trials on investigational products for human use (ENTR/CT3) and also in accordance with country-specific requirements.

The trial office shall notify the investigators of any AE associated with the use of investigational products in this study that is both serious and unexpected (i.e., SUSAR). The investigator must keep copies of all pertinent safety information on file including correspondence with the trial office and the ethics committee. (Cf. section 14.3 for record retention information).

12. DISCONTINUATIONS AND TREATMENT AFTER THE END OF TRIAL PARTICIPATION

12.1. Discontinuations

The following events are considered sufficient reasons for discontinuing a subject from the study:

- adverse event(s)
- disease progression
- withdrawal of consent
- patient preference
- death
- lost to follow up
- protocol violation

The reason for discontinuation should be recorded in the CRF and in the source documents.

12.2. Treatment after the End of Trial Participation

Patients completing treatment as stipulated in the protocol and patients discontinuing participation in the trial prematurely will be followed up as outlined in section 8.5. Treatment of refractory disease or relapse is at the discretion of the investigator. Although such treatment is not part of this protocol, the clinical course will be documented and the data provided to the trial

office.

13. REGULATORY CONSIDERATIONS

13.1. Good Clinical Practice

The procedures set out in this study protocol pertaining to the conduct, evaluation, and documentation of this study are designed to ensure that all investigators abide by Good Clinical Practice (GCP), as described in International Conference on Harmonization (ICH) Guideline E6 and in accordance with the general ethical principles outlined in the Declaration of Helsinki. The study will receive approval from an ethics committee prior to commencement. The investigator will conduct all aspects of this study in accordance with applicable national, state, and local laws of the pertinent regulatory authorities.

13.2. Investigator Responsibilities

Investigator responsibilities are set out in the ICH Guideline for Good Clinical Practice. The trial office will evaluate and approve all investigators who in turn will select their staff.

The investigator should ensure that all persons assisting with the study are adequately informed about the protocol, amendments, study treatments, as well as study-related duties and functions. The investigator should maintain a list of sub-investigators and other appropriately qualified persons to whom he or she has delegated significant study-related duties. The investigator is responsible for keeping a record of all subjects who sign an informed consent document (subject identification code list; Appendix 8).

The investigator, or a designated member of the investigator's staff, must be available during monitoring visits to review data, resolve queries and allow direct access to subject records (e.g., medical records, office charts, hospital charts, and study-related charts) for source data verification. The investigator must ensure timely and accurate completion of CRFs and queries.

13.3. Subject Information and Informed Consent

The investigator must inform the study subject of all alternative treatment options pertinent to his or her current disease state including high-dose chemotherapy with autologous blood stem cell transplantation which is considered the treatment of choice for patients below the age of 65 – 70 years able to tolerate the procedure. The investigator must obtain informed consent of the study subject prior to any study related procedures (cf. Appendix 5). Documentation that informed consent occurred prior to the study subject's entry into the study and of the informed consent process should be recorded in the study subject's source documents including the date. The original informed consent document signed and dated by the study subject and by the person consenting the study subject prior to the study subject's entry into the study, must be maintained in the investigator's study files and a copy given to the study subject. The investigator must inform the study subject that inclusion in the study requires the subject to consent that representatives of the trial office and, when necessary, representatives from regulatory authorities may review his or her coded study data. If a protocol is amended and it impacts on the content of the informed consent, the informed consent document must be revised. Study subjects participating in the study when the amended protocol is implemented must be re-consented with the revised version of the informed consent document. The revised informed consent document signed and dated by the study subject and by the person consenting the study subject must be maintained in the investigator's study files and a copy given to the study subject. The investigator must inform the study subject that participation in another study

requires approval by the coordinating principal investigator or trial coordinator.

13.4. Confidentiality

The trial office affirms the subject's right to protection against invasion of privacy and to be in compliance with ICH and other local regulations (whichever is most stringent). The trial office requires the investigator to permit representatives of the trial office and, when necessary, representatives from regulatory authorities, to review and/or copy any medical records relevant to the study in accordance with local laws.

Should direct access to medical records require a waiver or authorization separate from the subject’s signed informed consent document, it is the responsibility of the investigator to obtain such permission in writing from the appropriate individual.

13.5. Protocol Amendments

Amendments will be submitted to the ethics committee and regulatory authorities for written approval. Written approval must be obtained before implementation of the amended version occurs. The written signed approval from the ethics committee and regulatory authorities should specifically reference the investigator name, protocol number, study title and amendment number(s) that is applicable. Amendments that are administrative in nature do not require ethics committee approval but will be submitted to the ethics committee for information purposes.

13.6. Ethics Committee and Regulatory Authorities Review and Approval

Before the start of the study, the study protocol, informed consent document, and any other appropriate documents will be submitted to the ethics committee and the regulatory authorities with a cover letter listing the documents submitted, their dates of issue, and the sites for which approval is sought.

Lenalidomide can only be supplied to an investigator by Celgene or its authorized representative after documentation on all ethical and legal requirements for starting the study has been received by Celgene or its authorized representative. This documentation must also include a statement by the ethics committee confirming that the composition of the committee is in accordance with GCP. Formal approval by the ethics committee should mention the protocol title, number, amendment number (if applicable), study site, and any other documents reviewed. It must mention the date on which the decision was made and must be officially signed by a committee member. Before the first subject is enrolled in the study, all ethical and legal requirements must be met.

The ethics committee and the authorities must be informed of all subsequent protocol amendments in accordance with local legal requirements. Amendments must be evaluated to determine whether formal approval must be sought and whether the informed consent document should also be revised.

The trial office must keep a record of all communication with the ethics committee and the regulatory authorities.

13.7. Closure of the Study

The coordinating principal investigator reserves the right to terminate this study at any time for reasonable medical or administrative reasons. Any premature discontinuation will be

appropriately documented according to local requirements (e.g., ethics committee, regulatory authorities). In addition, the coordinating principal investigator has the right to discontinue a single site at any time during the study for medical or administrative reasons such as:

- unsatisfactory enrolment;
- GCP noncompliance;
- inaccurate or incomplete data collection;
- falsification of records;
- failure to adhere to the study protocol.

13.8. Study Subject Insurance

Patients participating in the LeMLAR trial will be protected against study-related damage by a study subject insurance with the HDI-GERLING Industrie Versicherung AG (police number 5701032803018). Patients will receive a copy of the insurance policy (cf. Appendix 7) together with the informed consent documents (cf. Appendix 5) and the lenalidomide information sheet (cf. Appendix 6).

13.9. Financial Aspects

Study subjects will not be recompensated for participation in the trial. Methotrexate, leucovorin, cytarabine and rituximab are approved for the treatment of B cell lymphomas. Treatment with these drugs will therefore be paid by the health insurance providers. Lenalidomide will be provided free of charge by Celgene. Treatment centers will be recompensated for documentation of treatment and outcome by the trial office.

14. DATA HANDLING AND RECORD KEEPING

14.1. Data/Documents

The investigator must ensure that the records and documents pertaining to the conduct of the study and the distribution of lenalidomide are complete, accurate, filed and retained. Examples of source documents include: hospital records; clinic and office charts; laboratory notes; memoranda; subject's diaries or evaluation checklists; dispensing records; recorded data from automated instruments; copies or transcriptions certified after verification as being accurate copies; microfiche; x-ray film and reports; and records kept at the pharmacy, and the laboratories, as well as copies of CRFs or CD-ROM.

14.2. Data Management

Data will be collected via CRF and entered into the clinical database of a clinical research organization to be appointed by and representing the trial office. This data will be electronically verified through use of programmed edit checks specified by the clinical team. Discrepancies in the data will be brought to the attention of the clinical team, and investigational site personnel, if necessary. Resolutions to these issues will be reflected in the database. An audit trail within the system will track all changes made to the data.

14.3. Record Retention

Essential documents must be retained by the investigator for a minimum of 10 years after the end of the trial period unless local laws or requirements dictate longer retention. Essential documents include, but are not limited to, the following:

- signed informed consent documents for all subjects;
- subject identification code list;
- record of all communications between the investigator and the trial office;
- list of sub-investigators and other appropriately qualified persons to whom the investigator has delegated significant study-related duties, together with their roles in the study, curriculum vitae, and their signatures;
- copies of CRFs (if paper) and of documentation of corrections for all subjects;
- lenalidomide accountability records;
- record of any body fluids or tissue samples retained;
- all other source documents (subject records, hospital records, laboratory records, etc.);
- all other documents as listed in Section 8 of the ICH consolidated guideline on GCP (Essential Documents for the Conduct of a Clinical Trial).

The investigator must notify the trial office if he/she wishes to assign the essential documents to someone else, remove them to another location or is unable to retain them for a specified period.

All study documents should be made available if required by relevant health authorities. The investigator should take measures to prevent accidental or premature destruction of these documents.

15. QUALITY CONTROL AND QUALITY ASSURANCE

All aspects of the study will be carefully monitored by the trial office or its authorized representative for compliance with applicable government regulations with respect to current GCP and standard operating procedures.

15.1. Study Monitoring and Source Data Verification

The coordinating principal investigator ensures that appropriate monitoring procedures are performed before, during and after the study. Before the study is initiated at a site visit or at an investigator meeting, all aspects of the study are reviewed with the investigator and the staff. Prior to enrolling subjects into the study, a representative of the trial office will review the protocol, CRFs, procedures for obtaining informed consent, record keeping, and reporting of AEs/SAEs with the investigator. Monitoring will include on-site visits with the investigator and his/her staff as well as any appropriate communications by mail, email, fax, or telephone. At each monitoring visit, the facilities, lenalidomide storage area, CRFs, subject's source documents, and all other study documentation will be inspected/reviewed by the trial office representative for accuracy, adherence to the protocol and Good Clinical Practice.

Accuracy will be checked by performing source data verification that is a direct comparison of the entries made onto the CRFs against the appropriate source documentation. Any resulting discrepancies will be reviewed with the investigator and/or his/her staff. Any necessary

corrections will be made directly to the CRFs or via queries by the investigator and/or his/her staff. Monitoring procedures require that informed consents, adherence to inclusion/exclusion criteria and documentation of SAEs and their proper recording be verified. Additional monitoring activities may be outlined in a study-specific monitoring plan.

15.2. Audits and Inspections

The investigator is required to permit direct access to the facilities where the study took place, source documents, CRFs and applicable supporting records of study subject participation for audits and inspections by authorized representatives of regulatory authorities. The investigator should make every effort to be available for the audits and/or inspections. If the investigator is contacted by any regulatory authority regarding an inspection, he/she should contact the trial office immediately.

16. PUBLICATIONS

The outline of this trial will be submitted for inclusion in the EudraCT and ClinicalTrials.gov databases. Its results may be presented at medical conferences and published in medical journals. Representatives of all trial sites actively recruiting patients will be included as authors. The coordinating principal investigator or trial coordinator will prepare a manuscript draft and send it to the co-authors and Celgene for internal review. The co-authors and Celgene need to notify the coordinating principal investigator or trial coordinator within 28 days of receipt of the manuscript of any changes to be made. The coordinating principal investigator or trial coordinator will decide whether the manuscript needs to be modified and subsequently submit it to a journal of his choice.

17. REFERENCES

1 Coiffier B et al. CHOP chemotherapy plus rituximab compared with CHOP alone in elderly patients with diffuse large-B-cell lymphoma. N Engl J Med 2002; 346: 235–242

2 Habermann TM et al. Rituximab-CHOP versus CHOP alone or with maintenance rituximab in older patients with diffuse large B-cell lymphoma. J Clin Oncol 2006; 24: 3121–3127

3 Sehn LH et al. Introduction of combined CHOP plus rituximab therapy dramatically improved outcome of diffuse large B-cell lymphoma in British Columbia. J Clin Oncol 2005; 23: 5027–5033

4 Pfreundschuh M et al. CHOP-like chemotherapy plus rituximab versus CHOP-like chemotherapy alone in young patients with goodprognosis diffuse large-B-cell lymphoma: a randomised controlled trial by the MabThera International Trial (MInT) Group. Lancet Oncol 2006; 7: 379–391

5 Feugier P et al. Long-term results of the R-CHOP study in the treatment of elderly patients with diffuse large B-cell lymphoma: a study by the Groupe d'Etude des Lymphomes de l'Adulte. J Clin Oncol 2005; 23: 4117-4126

6 Gisselbrecht C et al. Salvage regimens with autologous transplantation for relapsed large B-cell lymphoma in the rituximab era. J Clin Oncol 2010; 28: 4184-4190

7 Li S et al. Recent advances of IMiDs in cancer therapy. Curr Opin Oncol 2010; 22: 579-585

8 Wiernik PH et al. Lenalidomide monotherapy in relapsed or refractory aggressive non-Hodgkin's lymphoma. J Clin Oncol 2008; 26: 4952-4957

9 Witzig TE et al. An international phase II trial of single-agent lenalidomide for relapsed or refractory aggressive B-cell non-Hodgkin’s lymphoma. Ann Oncol 2011; 22: 1622-1627

10 Hernandez-Ilizaliturri FJ et al. Higher response to lenalidomide in relapsed(refractory diffuse large B-cell lymphoma in nongerminal center B-cell-like than in germinal center B-cell-like phenotype. Cancer 2011; 117: 5058-5066

11 Zinzani PL et al. Combination of lenalidomide and rituximab in elderly patients with relapsed and refractory diffuse large B-cell lymphoma: a phase 2 trial. Clin Lymphoma Myeloma Leuk 2011 May 4 [Epub ahead of print]

12 Nowakowski GS etal. Lenalidomide can be safely combined with R-CHOP (R2CHOP) in the initial chemotherapy for aggressive B-cell lymphomas: a phase I study. Leukemia 2011 July 1 [Epub ahead of print]

13 Levitt M et al. Combination sequential chemotherapy in advanced reticulum cell sarcoma. Cancer 1972; 29: 630-636

14 Gaynor ER et al. Treatment of diffuse histiocytic lymphoma (DHL) with COMLA (cyclophophamide, oncovin, methotrexate, leucovorin, cytosine arabinoside): a 10-year experience in a single institution. J Clin Oncol 1985; 3: 1596-1604

15 Fisher RI et al. Comparison of a standard regimen (CHOP) with three intensive chemotherapy regimens for advanced non-Hodgkin's lymphoma. N Engl J Med 1993; 328: 1002-1006

16 Lister TA et al. Report of a committee convened to discuss the evaluation and staging of patients with Hodgkin’s disease: Cotswolds Meeting. J Clin Oncol 1989; 7: 1630-1636

17 A predictive model for aggressive non-Hodgkin's lymphoma. The International Non-Hodgkin's Lymphoma Prognostic Factors Project. N Engl J Med 1993; 329: 987-994

18 Cockcroft DW, Gault MH. Prediction of creatinine clearance from serum creatinine. Nephron 1976; 16: 31-41

19 Cheson BD et al. Report of an international workshop to standardize response criteria for non-Hodgkin’s lymphomas. J Clin Oncol 1999; 17: 1244-1253

20 Fleming TR. One-sample multiple testing procedure for phase II clinical trials. Biometrics 1982; 38: 143-151

21 A’Hern RP. Sample size tables for exact single-stage phase II designs. Stat Med 2001; 20: 859-866
